# Identification of immune-related ferroptosis prognostic marker and in-depth bioinformatics exploration of multi-omics mechanisms in thyroid cancer

**DOI:** 10.3389/fmolb.2022.961450

**Published:** 2022-08-17

**Authors:** Xin Fan, Fei Xie, Lingling Zhang, Chang Tong, Zhiyuan Zhang

**Affiliations:** ^1^ Department of Otolaryngology-Head and Neck Surgery, The First Affiliated Hospital of Nanchang University, Nanchang, China; ^2^ School of Stomatology, Nanchang University, Nanchang, China; ^3^ Pediatric Medical School, Nanchang University, Nanchang, China

**Keywords:** thyroid cancer, immune-related ferroptosis, prognostic marker, multi-omics mechanisms, bioinformatics exploration

## Abstract

**Background:** Factors such as variations in thyroid carcinoma (THCA) gene characteristics could influence the clinical outcome. Ferroptosis and immunity have been verified to play an essential role in various cancers, and could affect the cancer patients’ prognosis. However, their relationship to the progression and prognosis of many types of THCA remains unclear.

**Methods:** First, we extracted prognosis-related immune-related genes and ferroptosis-related genes from 2 databases for co-expression analysis to obtain prognosis-related differentially expressed immune-related ferroptosis genes (PR-DE-IRFeGs), and screened BID and CDKN2A for building a prognostic model. Subsequently, multiple validation methods were used to test the model’s performance and compare its performance with other 4 external models. Then, we explored the mechanism of immunity and ferroptosis in the occurrence, development and prognosis of THCA from the perspectives of anti-tumor immunity, CDKN2A-related competitive endogenous RNA regulatory, copy number variations and high frequency gene mutation. Finally, we evaluated this model’s clinical practice value.

**Results:** BID and CDKN2A were identified as prognostic risk and protective factors, respectively. External data and qRT-PCR experiment also validated their differential expression. The model’s excellent performance has been repeatedly verified and outperformed other models. Risk scores were significantly associated with most immune cells/functions. Risk score/2 PR-DE-IRFeGs expression was strongly associated with BRAF/NRAS/HRAS mutation. Single copy number deletion of CDKN2A is associated with upregulation of CDKN2A expression and worse prognosis. The predicted regulatory network consisting of CYTOR, hsa-miRNA-873-5p and CDKN2A was shown to significantly affect prognosis. The model and corresponding nomogram have been shown to have excellent clinical practice value.

**Conclusion:** The model can effectively predict the THCA patients’ prognosis and guide clinical treatment. Ferroptosis and immunity may be involved in the THCA’s progression through antitumor immunity and BRAF/NRAS/HRAS mutation. CYTOR-hsa-miRNA-873-5p-CDKN2A regulatory networks and single copy number deletion of CDKN2A may also affect THCA′ progression and prognosis.

## Introduction

Thyroid carcinoma (THCA) is one of the most common endocrine carcinomas, and its morbidity has increased steadily over the last 3 decades (Filetti et al., 2019). In the U.S., an estimated 44,280 new THCA cases and 2,200 new THCA deaths are expected in 2021 (Siegel et al., 2021). The choice of treatment method for THCA is closely related to its differentiation degree and pathological type (Wallner et al., 2019). Traditional treatments include thyroidectomy, radioactive iodine therapy and endocrine therapy (Schlumberger and Leboulleux, 2021). Unfortunately, 15–20% of differentiated THCA and most anaplastic THCA patients have poor clinical outcomes (Naoum et al., 2018). And treatments for advanced THCA are limited (Mehnert et al., 2019). As a new era in cancer treatment (Ngamcherdtrakul et al., 2021; Johnson et al., 2022), immune checkpoint inhibitors (ICIs) has achieved positive efficacy in many tumors such as non-small cell lung cancer (NSCLC) (Kazandjian et al., 2016), melanoma (Bhatnagar et al., 2017), head and neck squamous cell carcinoma (Larkins et al., 2017). Similarly, it has broad prospects in the clinical treatment of THCA (Varricchi et al., 2019; Di Molfetta et al., 2021). While ICIs can significantly improve survival in several cancers, they may also induce a series of immune-related adverse events (irAEs) with endocrine disorders being the most common (Wright et al., 2021). Thyroid becomes the endocrine organ most commonly affected by ICIs, which usually results in hypothyroidism and thyrotoxicosis (Wright et al., 2021). Therefore, it is recommended to perform thyroid function tests every 6–12 weeks at baseline, before each dose, and every 6–12 weeks for the first 6 months after completion of treatment to scientifically manage potential irAEs (González-Rodríguez and Rodríguez-Abreu, 2016).

Ferroptosis is an iron-dependent form of cell death distinguished from other programmed cell death, which was put forward in 2012 (Dixon et al., 2012). Ferroptosis is closely related to metabolic events in cells driven by lipid peroxidation, playing an important role in cancer progression (Stockwell et al., 2020; Jiang et al., 2021). The ferroptosis process in tumors is observed to be closely related to the immune microenvironment, which also implicates frequent collaboration of ferroptosis and immunity in tumor progression (Stockwell et al., 2020; Jiang et al., 2021). Many studies have reported that ferroptosis inducers can enhance the efficacy of ICIs immunotherapy (Friedmann Angeli et al., 2019; Song et al., 2021; Xu et al., 2021). In addition, ferroptosis-related genes (FRGs) are closely related to tumor immunity and chemotherapy resistance and can be used as indicators of clinical prognosis in tumor patients (Turubanova et al., 2019; Yu et al., 2020). Competitive endogenous RNAs (ceRNA) is an important issue in recent years. Long non-coding RNA (LncRNA) adsorbs microRNA (miRNA) to regulate gene expression, which plays an important role in the occurrence and development of tumors and other diseases (Karreth and Pandolfi, 2013; Chan and Tay, 2018). Copy number variation (CNV) refers to an increase or decrease in the copy number of large segments of the genome, ranging in size from approximately 1 KB to 3 Mb, which may occur at coding gene sites or be associated with signaling pathways such as cell proliferation, chromosome replication, and repair (Carson et al., 2006). CNV changes the expression level of its encoded product and thus changes the occurrence and development of tumors (Liu et al., 2012; Jiang et al., 2014; Yang et al., 2014).

The above results all indicate that ferroptosis and immunity have great potential value in tumor progression as well as in predicting prognosis and clinical treatment effect. Therefore, it is necessary to screen the prognosis-related differentially expressed immune-related ferroptosis genes (PR-DE-IRFeGs) by bioinformatics to construct marker that can effectively predict THCA patients’ prognosis and clinical treatment effect. Through multi-omics analysis, the exploration of the mechanism of immunity and ferroptosis in the occurrence, development and prognosis of THCA from the aspects of anti-tumor immunity, CDKN2A-related ceRNA regulation, CNV and high-frequency gene mutation will also provide many scientific values.

## Material and methods

### Extraction of samples and data

**FIGURE 1:**
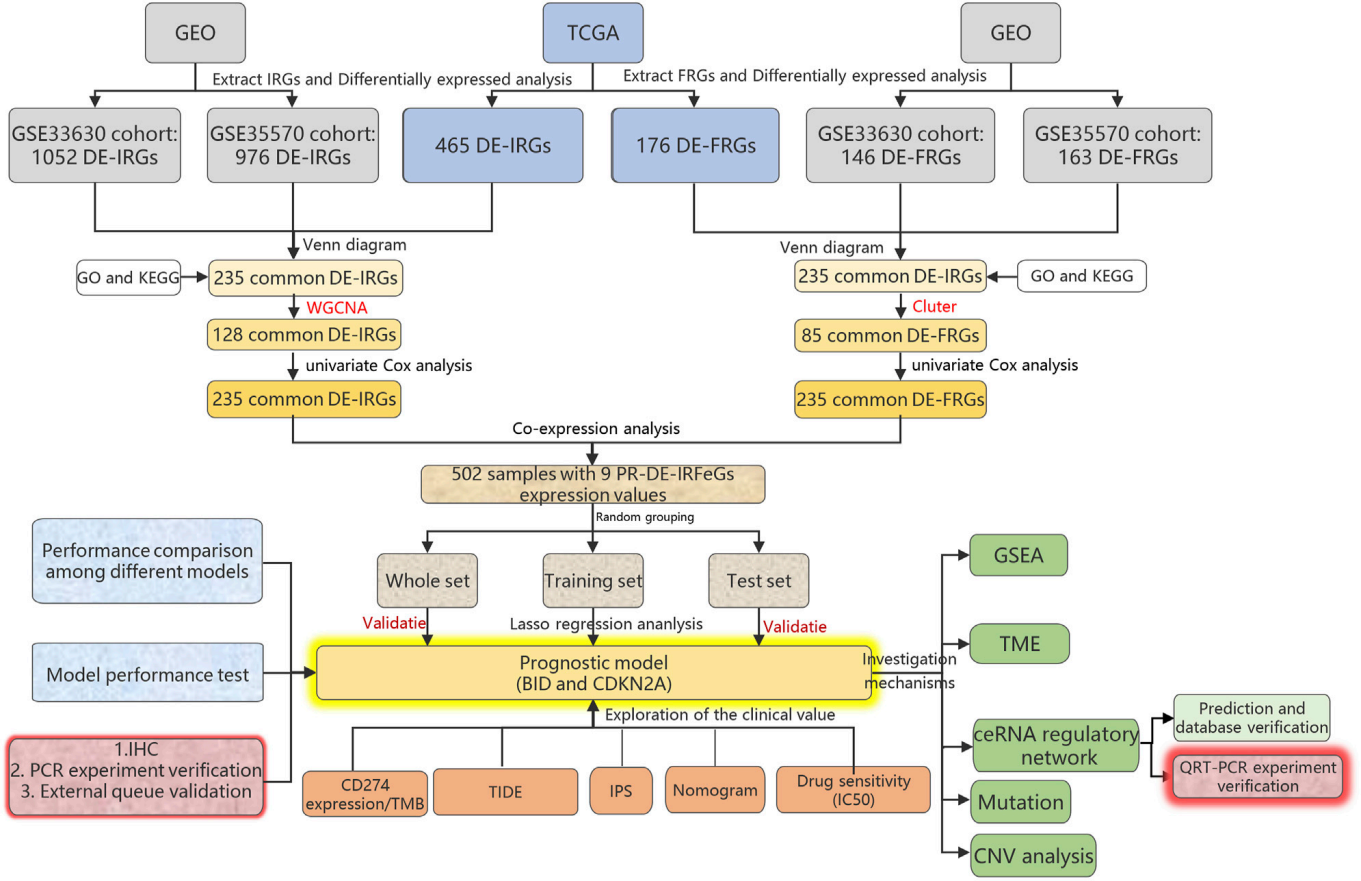
Research diagram of the whole process.

The overview of our research process was shown in Figure 1. On 24 November 2021, we obtained THCA samples and data from 2 databases and 3 public cohorts. We first extracted RNA sequencing and corresponding clinical data of 568 samples (510 THCA and 58 adjacent normal tissues) using The Cancer Genome Atlas database (TCGA, cancergenome.nih.gov/) on 24 November 2021. We then obtained two other external cohorts, the GSE33630 and GSE35570 cohorts, from the Gene Expression Omnibus (GEO) database (ncbi.nlm.nih.gov/geo/) on 24 November 2021. The former contained 105 samples (49 papillary thyroid carcinomas, 11 anaplastic thyroid carcinomas, and 45 normal thyroids) and the latter 116 samples (65 papillary thyroid carcinomas and 51 normal thyroids). Finally, we successfully extracted 2660 immune-related genes (IRGs) from the ImmPort (immport.org/home) and InnateDB (innatedb.ca/) databases on 31 July 2021.259 FRGs were extracted from the FerrDb (zhounan.org/ferrdb) database on 31 July 2021.

### Identification of DE-IRGs and DE-FRGs

After overlapping IRGs list, FRGs list and RNA sequencing data of TCGA, GSE33630 and GSE35570 cohorts, the RNA sequencing data of 2365 IRGs and 246 FRGs were obtained from TCGA cohorts. The RNA sequencing data of 1879 IRGs and 234 FRGs were obtained from the GSE33630 cohort, and RNA sequencing data of 1879 IRGs and 234 FRGs were obtained from the GSE35570 cohort.

We used the R package “limma” to explore 510 THCA and 58 adjacent normal tissues in the TCGA cohort for differentially expressed immune-related genes (DE-IRGs) based on the filter conditions |log2 fold change| (|log2FC|) >1 and false discovery rate (FDR) < 0.05. For RNA sequencing data of DE-IRGs from 2 GEO cohorts and differentially expressed ferroptosis-related genes (DE-FRGs) from the three cohorts, FDR <0.05 became a new filter condition. The R “Venn” was used to describe the crossover results of DE-IRGs/DE-FRGs for the 3 cohorts.

### GO and KEGG enrichment analysis based on the common DE-IRGs and DE-FRGs

We used bubble chart and histograms to demonstrate the pathways and functions of enrichment in the common DE-IRGs and common DE-FRGs. To do so, the R package “org.Hs.eg.db” was used to perform the Kyoto Encyclopedia of Genes and Genomes (KEGG) and Gene Ontology (GO).

### Identification of PR-DE-IRFeGs

After obtaining the expression data profile of common DE-IRGs based on 3 cohorts, we used the R package “weighted gene expression network analysis (WGCNA) " to identify DE-IRGs highly correlated to THCA. The TCGA samples were clustered to eliminate the free samples, and then the function pickSoftThreshold was used to select the best soft power β = 6 to build the best scale-free network. First, we created the adjacency matrix according to the formula:

$$a_{\mathrm{ij}}={\left|S_{\mathrm{ij}}\right|}_{\beta }$$

(a_ij_: adjacency matrix between gene i and gene j, S_ij_: similarity matrix which is done by Pearson correlation of all gene pairs, β: softpower value) (Yip and Horvath, 2007). Next, it was transformed into a topological overlap matrix (TOM) and the corresponding dissimilarity (1-TOM) (Yip and Horvath, 2007). The genes were clustered at a distance of 1-TOM to construct corresponding modules for matching corresponding dynamic branches (Lombardo et al., 2018). After similar modules were merged, two modules were obtained from TCGA cohort. Similarly, we identified two modules from GSE33630 cohort with the best soft power β = 8 and GSE35570 cohort with the best soft power β = 7, respectively. Meanwhile, genes in modules with the highest correlation coefficients from 3 cohorts were extracted for common DE-IRGs highly correlated to THCA.

R package “ConsensusClusterPlus” was used to run the cluster analysis (1000 iterations and 80% resampled rate) for identifying ferroptosis-associated molecules subtypes based on 110 DE-FRGs’ RNA sequencing data, and to classify THCA patients into different subtypes. Kaplan-Meier survival curves were used to compare overall survival (OS) between the two subtypes. Likewise, differential expression analysis of 110 DE-FRGs between the two subtypes was performed to identify differentially expressed DE-FRGs according to a filter criterion of FDR <0.05. We also visualized the differences in clinicopathological features and expression of these DE-FRGs between the two subtypes with a heatmap.

*p* < 0.05 was used as filter criterion for univariate cox regression based on common DE-IRGs highly correlated to THCA/differentially expressed DE-FRGs expression and OS from TCGA cohort.

Finally, 35 DE-IRGs and 10 DE-FRGs with prognostic values were extracted. Then, we set the threshold of correlation coefficient >0.3 and *p* value < 0.001 for co-expression analysis between their expression values to filter the corresponding PR-DE-IRFeGs.

### Construction and verification of prognostic model

We randomly matched 502 samples with complete OS data to the training and test sets with a ratio of 5:5. The expression values of the 9 prognostic DE-IRFeGs, the survival data of all samples in the training set were used to run lasso regression and multivariate Cox regression. After constructing the final multivariate Cox regression model, the risk score of each sample was based on the following formula calculates:

Risk score=∑(PR−DE−IRFeGs expression values×corresponding coefficient)

All cases from the TCGA database obtained their risk scores. Cases from total, training and test sets were divided into high-risk and low-risk groups using the median risk score of each set as the cutoff point, respectively.

We used the R tool to draw the risk curves and survival status diagrams to visualize the relationship between the risk score and survival status of each case, and created a Kaplan-Meier curve to clarify the correlation between the risk groups and OS. The expression distribution of the 2 PR-DE-IRFeGs with increasing risk scores was also visualized. Receiver operating characteristic curve (ROC) curves with area under the curve (AUC) for response prediction accuracy was drawn to verify the model’s prediction accuracy based on R package “timeROC”. Univariate and multivariate Cox regressions were performed to verify whether our model could independently predict patients’ OS under multifactorial clinical conditions.

### Comparison of performance among different prognostic models

To compare the ability of our model to predict prognosis with other models, we selected four models constructed by Han et al. (2020), Wu et al. (2021), Huang et al. (2021), and Qian et al. (2021). These models are based on 4 pyroptosis-related genes (IL18, GSDMC, PJVK, and NOD1), 3 ferroptosis-related genes (HSPA5, AURKA, and TSC22D3), 5 ferroptosis-related genes (DPP4, GSS, HMGCR, PGD, and TFRC) and 5 autophagy-related genes (CX3CL1, ATG9B, CDKN2A, ITPR1, and DNAJB1), respectively. The messenger RNA (mRNA) expression data corresponding to the genes in each model was extracted from the TCGA THCA cohort. These data were used to construct a multiCox regression model to calculate risk scores for individual samples. We then divided the samples into high-risk and low-risk groups based on their median risk scores. ROC curves and Kaplan-Meier survival curves were used to compare the performance of five models in predicting prognosis and the ability to distinguish prognosis, respectively. The mRNA expression levels of the genes corresponding to these four models and our prognostic model were used for calculating and comparing the concordance index (C-index). This process was performed based on R package “survcomp”. In addition, we used restricted mean survival time (RMST) curves to evaluate the performance of each model and compare their differences (Schröder et al., 2011; Zheng et al., 2021).

### Verification of differential expression of 2 PR-DE-IRFeGs protein levels

By comparing the immunohistochemical (IHC) staining images of BID and CDKN2A, we obtained the results of their differential expression between THCA tissue and normal thyroid tissue. IHC staining images were provided by the Human Protein Atlas (HPA) database (proteinatlas.org) on 25 November 2021.

### Model performance evaluation in clinical subgroups

The results of the stratified analysis can show whether this model can still have advantages in different subgroups of clinicopathological features. The TCGA samples could be divided into different subgroups due to differences in 6 clinical parameters. Kaplan-Meier analysis clearly showed differences in survival probability between different risk groups in each subgroup.

### GSEA enrichment analysis among different risk groups

Differentially expressed genes between high and low risk groups were used in the enrichment analysis of biological functions and pathways involved in the high and low risk groups based on the R package “cluster profile” and the gene sets “c2. cp.kegg.v7.4. symbols.gmt”, “c5. go.v7.4. symbols.gmt".

### Immune cell and function analysis

Through Gene Set Enrichment Analysis (GSEA, broad.mit.edu/GSEA) enrichment analysis on 26 November 2021, numerous functions and pathways related to immunity were obtained. We then used single-sample gene set enrichment analysis (ssGSEA) to quantify each sample’s immune cells and functions scores for correlation and difference analysis with risk scores. ssGSEA was done by R packages “GSEAbase” and “gsva”. Such results were visualized by heat maps, showing their distribution differences in each sample.

### Prediction of a network regulatory network targeting CDKN2A

To further investigate the regulatory mechanism of CDKN2A related to lncRNAs and miRNAs, we obtained RNA sequencing data of miRNAs and mRNAs from TCGA for analysis. First, a RNA sequencing package of CDKN2A across 33 types of cancer was downloaded from the UCSC Xena (xena.ucsc.edu) database on 14 January 2022. These RNA-sequencing data were used to distinguish differential expression of CDKN2A between cancer and adjacent tissues. Then, we acquired the miRNA expression data of 573 samples from TCGA, including 514 THCA and 59 adjacent normal tissues for the next analysis. Candidate miRNAs bound the upstream of CDKN2A were predicted using various programs in StarBase (starbase.sysu.edu.cn) for correlation analysis with CDKN2A expression (threshold: correlation coefficient < -0.288, *p* < 0.001) on 15 January 2022. To clarify differentially expressed miRNAs between THCA and normal cases, a differential analysis (|log2FC|) >1, *p* < 0.05) was used. Survival probability between subgroups of cases with different miRNA expression was also compared. No candidate miRNAs other than hsa-miR-873-5p showed significant significance in all results for further analysis. LncRNAs bound to hsa-miR-873-5p were predicted using StarBase (v2.0). Likewise, correlations between expression levels of lncRNAs and hsa-miR-873-5p (threshold: correlation coefficient < −0.29, *p* < 0.001)/CDKN2A (threshold: correlation coefficient < −0.48, *p* < 0.001), as well as the difference (| log2FC |) >1, *p* < 0.05) and survival (*p* < 0.05) analysis of lncRNAs were analyzed. Only CYTOR was considered statistically significant. Eventually, the ceRNA regulatory network made up of CYTOR, hsa-miR-873-5p and CDKN2A was visualized by Cytoscape (v3.7.2). To show the targeting relationship between the two genes in ceRNA for more detail, we also showed the predicted binding sites of hsa-miR-873-5p and CDKN2A as well as hsa-miR-873-5p and CYTOR through the Starbase website.

### Somatic gene mutation related to the model

VarScan was used to detect MAF files of somatic gene mutation data downloaded from the TCGA cohort for THCA samples, and we then used the R package “GenVisR” to visualize the 30 most frequently mutated genes in different risk groups, respectively. Perl was used to calculate each sample’s tumor mutation burden (TMB) for correlation analysis with risk scores and difference analysis between different risk groups. We compared the differences in risk score/BID/CDKN2A/CDKN2A-related DE-IRGs/BID-related DE-IRGs expression between BRAF/NRAS/HRAS wild and mutant groups, respectively.

### CNV analysis

We obtained CNV data for 9 PR-DE-IRFeGs of 512 THCA samples from the UCSC Xena database for our analysis. The analysis mainly included counting and visualizing the CNV frequencies of these genes and their chromosomal locations. For further analysis, the entire samples were split into normal, and single-gain/single-deletion copy groups according to variants of copy numbers in BID and CDKN2A. Then, the differential expression of PR-DE-IRFeGs between 2 groups was assessed. Additionally, survival differences between 2 groups were analyzed by constructing Kaplan-Meier survival plots.

### Treatment value of the model

Programmed death-ligand 1 (PD-L1 or CD274) and TMB, indicators for predicting the outcome of immunotherapy, were further analyzed. We used a circle diagram to demonstrate the relationship between TMB/CD274 expression and risk score/BID/CDKN2A expression. For prediction of the potential response of risk score to immune checkpoint block therapy, the Tumor Immune Dysfunction and Exclusion (TIDE) algorithm was also applied (Banchereau et al., 2021). Then, spearman correlation analysis was performed to reveal the relationship between TIDE, Microsatellite Instability (MSI), Dysfunction, Exclusion and risk score/BID/CDKN2A expression with a circle graph showing the results. Also, the comparison of CD274 expression, TMB, TIDE, MSI, Dysfunction and Exclusion scores among different risk groups was made.

Further, immunogenicity was analyzed by using the immunophenotypic score (IPS) data acquired from the Cancer Immunology Atlas (TCIA, tcia.at/home) database on 28 November 2021. We calculated the IPS of the samples by analyzing the gene expression levels of the 4 cell types that determined immunogenicity, which is proportional to the IPS score (Wang. et al., 2021a). The relationship between IPS and the risk score/BID/CDKN2A expression was explored by using the spearman correlation. The violin plots to graphically show the differences in IPS of the 3 types among different risk groups.

Finally, we analyzed the half-maximal inhibitory concentration (IC50) of 6 chemotherapeutics for THCA patients from the NCCN guidelines. The R package “pRophetic” was performed to predict IC50 with the total cohort of samples. Then a regression model based on the cell line expression data from the Genomics of Drug Sensitivity in Cancer (GDSC, cancerrxgene.org) database and the RNA sequencing transcriptome data from the TCGA database was constructed to predict the IC50 of drugs in each sample on 28 November 2021 (Kim et al., 2021). Spearman correlation analysis was used to explore the relationship between the IC50 of the 6 drugs and the risk score/BID/CDKN2A expression. Also, a comparison of the IC50 difference among different risk groups was performed.

### Combination of a nomogram for prediction of survival probability

Quantitatively predicting and monitoring the prognosis of THCA patients is essential for clinical practice. To do so, we integrated age, clinical stage and risk group to construct a nomogram by running the R package “rms”. ROC curves for 1-, 2-, 3-, 4- and 5- years were plotted to evaluate the prediction performance of the nomogram. Further, the accuracy of predicting the nomogram’s survival rate was tested using the calibration curve.

### Validation of differential expression of model genes using an external cohort

The GSE60542 cohort of the GEO database was used as an external cohort, from which we obtained RNA-sequencing data for 27 paired THCA and adjacent normal thyroid tissue. We used paired *t*-test to compare the differences of 2 PR-IRFeGs between THCA and adjacent normal thyroid tissue.

### Quantitative real-time polymerase chain reaction (qRT-PCR) to verify the relative expression differences of related genes

Twenty pairs of matched THCA tissues and adjacent normal thyroid tissues were obtained from patients undergoing surgical treatment at the First Affiliated Hospital of Nanchang University from September 2021 to April 2022. The collection of these tissues has been approved by the Ethics Committee of the First Affiliated Hospital of Nanchang University (2021-09-024) and written informed consents were obtained from all patients. We immediately immersed the removed tissue in RNA preservation solution (Servicebio, Wuhan, China) and stored in a −80°C freezer. QRT-PCR was used to detect the relative mRNA expression of genes.

Total RNA extracted from tissues using the TransZol Up Plus RNA Kit (TRANS, Beijing, China) was reverse transcribed into cDNA using EasyScript First-Strand cDNA Synthesis SuperMix (TRANS, Beijing, China). After amplification and detection of BID, CDKN2A and CYTOR using the Archimed Quantitative PCR Detection System and PerfectStart^®^ Green qPCR SuperMix (TRANS, Beijing, China), the detected values of the three genes were normalized to the relative expression values of β-actin by the 2^−ΔΔCt^ method. miRNAs extracted from tissues using the MiPure Cell/Tissue miRNAKit (Vazyme, Nanjing, China) was reverse transcribed into cDNA using miRNA first Strand cDNA Synthesis Kit (Vazyme, Nanjing, China). After amplification and detection of hsa-miR-873-5p using the Archimed Quantitative PCR Detection System and miRNA Universal SYBRqPCR Master Mix (TRANS, Beijing, China), the detected values of hsa-miR-873-5p were normalized to the relative expression values of U6 by the 2^−ΔΔCt^ method.

We ran paired t-tests to compare differences in relative expression of these genes between paired tissues. Supplementary Table S1 presented the primer sequences for all genes.

### Statistical processing

Throughout the process, the statistical methods used were portrayed below. In all statistical methods, results at *p* < 0.05 were considered statistically significant unless otherwise stated. We used the student’s t-test or non-parametric test to compare differences between continuous variables and the chi-square test or Fisher’s exact test to compare differences between categorical variables (Fan et al., 2021). Prognosis-related differentially expressed immune-related genes (PR-DE-IRGs) and prognosis-related differentially expressed ferroptosis-related genes (PR-DE-FRGs) were identified using univariate cox regression analysis. Lasso regression and multiCox regression were utilized to figure out PR-DE-IRFeGs to construct the prognostic model. The difference in Survival Probability between subgroups were compared using Kaplan-Meier analysis and log-rank test. Univariate and multivariate Cox analyses based on each clinical feature and risk score were used to verify the independent prognostic value of risk score (Fan et al., 2021). Correlation between variables was examined by Spearman or Pearson correlation analysis. All analyses were done using the R programming language (version 4.0.3), Perl (version 5.6.1) and Cytoscape (version v3.7.2).

## 3 Results

### Identification of DE-IRGs and DE-FRGs

**FIGURE 2:**
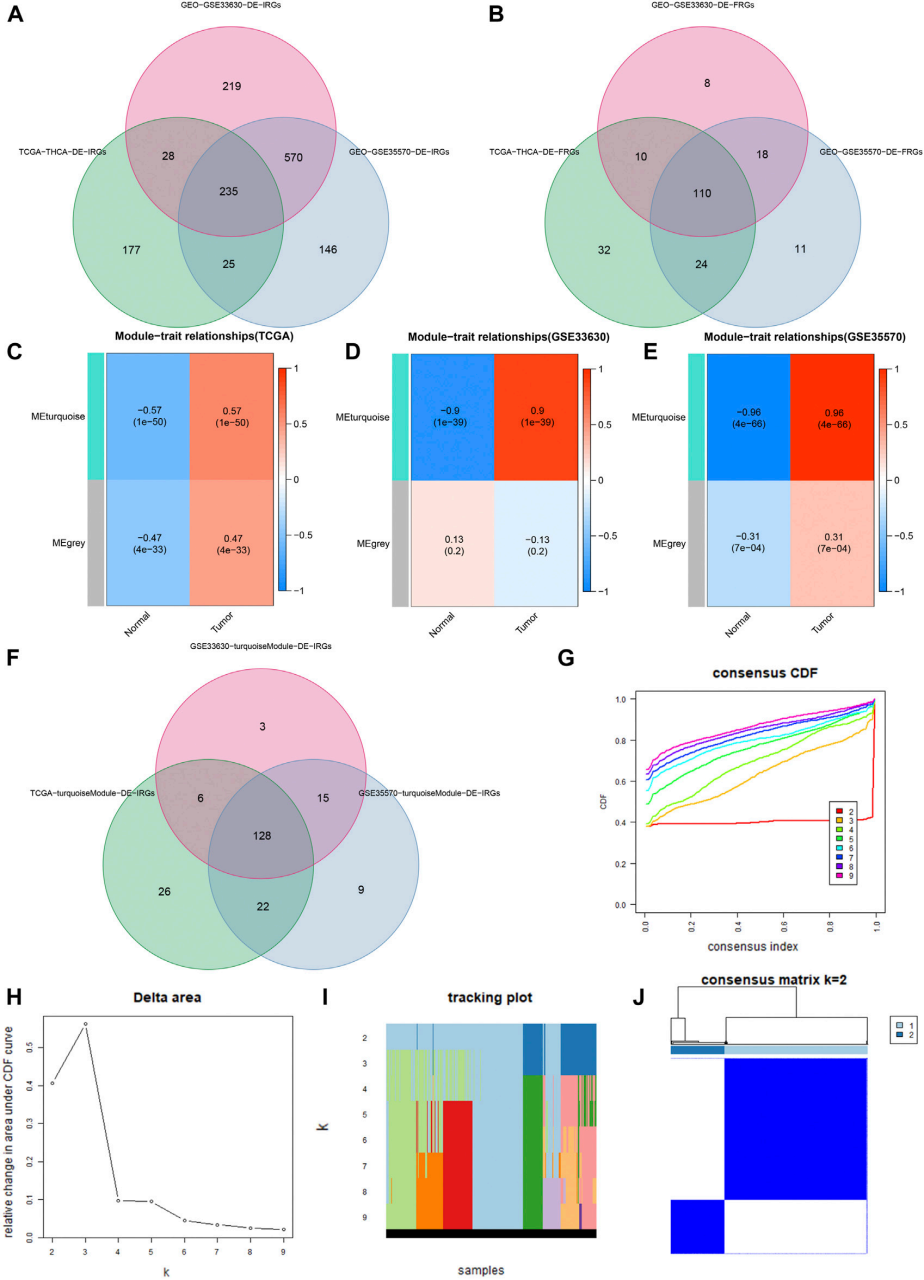
Identification of DE-IRGs and DE-FRGs. **(A)** Gene Venn plot based on DE-IRGs of the three cohorts. **(B)** Gene Venn plot based on DE-FRGs of the three cohorts. **(C–E)** Heatmaps of correlations between modules and tumor features in the TCGA, GSE33630, and GSE35570 cohorts, respectively. Each row corresponds to a color module, the left column represents the normal tissue, and the right column represents the tumor tissue. **(F)** DE-IRGs were obtained by the intersection of three cohorts’ turquoise modules. **(G)** Consensus cluster cumulative distribution function (CDF) for k = 2 to 9. **(H)** Relative change in the area under the CDF curve from k = 2 to 9. **(I)** Trace plots from k = 2 to 9. **(J)** Consensus clustering matrix in k = 2.

The DE-IRGs and DE-FRGs that we got are as follows: 465 DE-IRGs (230 genes: down-regulated; 235 genes: up-regulated) from the TCGA cohort, 1052 DE-IRGs from the GSE33630 cohort (610 genes: down-regulated; 442 genes: up-regulated), 976 DE-IRGs from the GSE35570 cohort (399 genes: down-regulated; 577 genes: up-regulated), 176 DE-FRGs from the TCGA cohort (98 genes: down-regulated; 78 genes: up-regulated), 146 DE-FRGs (90 genes: down-regulated; 56 genes: up-regulated) from the GSE33630 cohort, and 163 DE-FRGs (101 genes: down-regulated; 62 genes: up-regulated) were from the GSE35570 cohort. Finally, we found 235 DE-IRGs and 110 DE-FRGs shared by all three cohorts (Figures 2A,B).

### GO and KEGG enrichment analysis based on the common DE-IRGs and DE-FRGs

Supplementary Figure S1A shows that the screened DE-IRGs-enriched biological processes (BPs) were mainly associated with immune cell infiltration, such as negative regulation of cell adhesion, myeloid leukocyte migration, leukocyte chemotaxis, monocyte chemotaxis. Further KEGG analysis found that these genes were mainly enriched for cytokine-cytokine receptor interaction, MAPK signaling pathway, chemokine signaling pathway, and calcium signaling pathway (Supplementary Figure S1B). These pathways are closely related to inflammatory cell chemotaxis, further suggesting that inflammatory cell chemotaxis is closely related to the DE-IRGs we screened.

According to the results of the study (Fuhrmann et al., 2020; Fu et al., 2021), hypoxia can protect the effects in the ferroptosis process of tumor cells, and reduce out of ferroptosis inducers. In the absence of hypoxia, iron production is increased, and reducing stable iron protein production, which shows that hypoxia and ferroptosis have a very close relationship (Huo et al., 2021). In the GO analysis of DE-FRGs, the BPs enriched by these genes were closely related to the hypoxia response, mainly including response to oxidative stress, cellular response to chemical, stress, cellular response to oxidative stress, response to nutrient levels, response to extracellular stimulus, intrinsic apoptotic signaling pathway, etc. (Supplementary Figure S1C). As shown in Supplementary Figure S1D, these genes were mainly enriched for ferroptosis, autophagy, chemical carcinogenesis, FoxO signaling pathway, NOD−like receptor signaling pathway, Mitophagy, HIF−1 signaling pathway, etc. in KEGG analysis. These pathways are closely related to hypoxia, suggesting that ferroptosis has a strong correlation with the DE-FRGs genes we screened.

### Acquisition of PR-DE-IRFeGs

**FIGURE 3:**
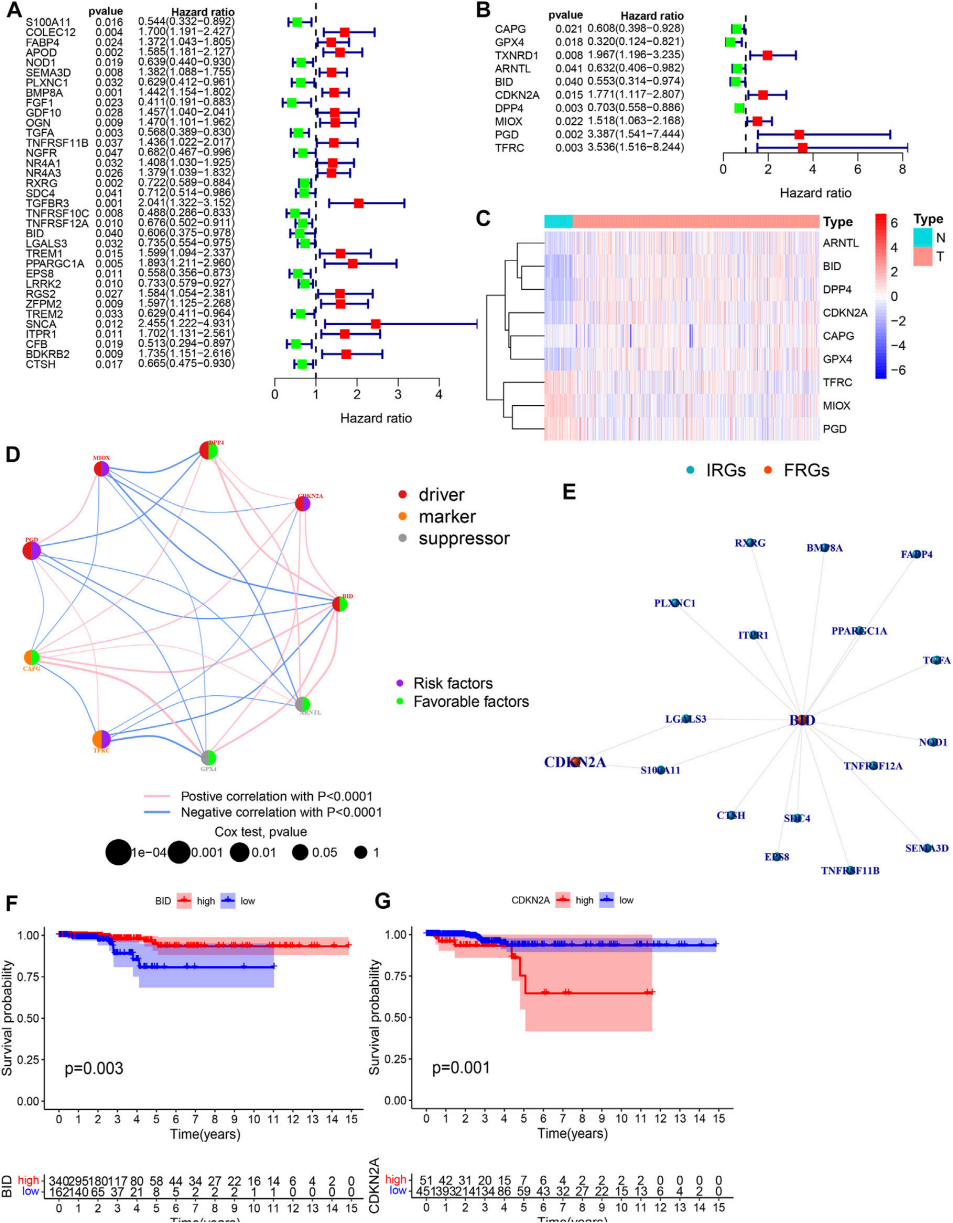
Acquisition of PR-DE-IRFeGs. **(A,B)** Univariate Cox regression analysis’ forest plot based on 35 PR-DE-IRGs and 10 PR-DE-FRGs, respectively. **(C)** Heatmap constructed based on the expression status of nine PR-DE-IRFeGs in tumor and normal samples. **(D)** Correlation network among 9 PR-DE-IRFeGs. The left half of the circle represents the properties of the gene, and the right half represents the gene’s effect on prognosis. **(E)** Co-expression network between CDKN2A/BID and their corresponding DE-IRGs. **(F,G)** Survival curves were constructed based on CDKN2A and BID genes, respectively.

We used WGCNA to build gene co-expression networks for the 235 common DE-IRGs in these 3 cohorts to identify the gene modules highly correlated to THCA. We obtained 2 modules in each of the 3 cohorts. Figures 2C–E shows the correlation results between these modules and THCA/normal tissue. Among these modules, three had the highest positive correlation with THCA and they were the turquoise modules in the TCGA cohort, the GSE33630 cohort, and the GSE35570 cohort. 128 common DE-IRGs in these 3 modules can be obtained by the Venn diagram (Figure 2F). The similarity in expression levels and the proportion of ambiguous clustering measurements we measured for the 110 DE-FRGs shared by the three cohorts ultimately determined k = 2 to have the best cluster stability (Figures 2G–I). All THCA patient samples (*n* = 502) were divided into two clusters, cluster1 (n = 364) and cluster2 (*n* = 138) (Figure 2J). Differences in the distribution of various clinicopathological features in the two clusters were visualized by the heatmap (Supplementary Figure S2A). It is also evident that samples in cluster2 had lower survival rates than in cluster1 (Supplementary Figure S2B). Therefore, 85 DE-FRGs were screened. The samples (*n* = 502) we got from the TCGA cohort for mRNA expression and clinical data whose clinical characteristics were shown in Table 1. Finally, 35 PR-DE-IRGs and 10 PR-DE-FRGs were screened by using univariate cox analysis, respectively (Figures 3A,B). Co-expression analysis of these RNA data resulted in 9 PR-DE-IRFeGs. Their expression distributions were shown with a heatmap (Figure 3C), and their relationships was visualized in a network graph (Figure 3D). Furthermore, it can be concluded that the risk factors considered prognostically related are CDKN2A, MIOX, PGD, and TFRC, while CAPG, GPX4, ARNTL, BID and DPP4 are considered protective factors (Figures 3A,B).

**TABLE 1:** Clinical characteristics of each set from TCGA cohort.

Covariates	Type	Total set	Test set	Training set	*p* value
Survival time (day)	≤712	246 (49%)	123 (49%)	123 (49%)	1
	>712	256 (51%)	128 (51%)	128 (51%)	
Survival state	Alive	486 (96.81%)	245 (97.61%)	241 (96.02%)	0.4459
	Deceased	16 (3.19%)	6 (2.39%)	10 (3.98%)	
Age (year)	≤60	389 (77.49%)	195 (77.69%)	194 (77.29%)	1
	>60	113 (22.51%)	56 (22.31%)	57 (22.71%)	
Gender	FEMALE	367 (73.11%)	180 (71.71%)	187 (74.5%)	0.5459
	MALE	135 (26.89%)	71 (28.29%)	64 (25.5%)	
Stage	I	281 (55.98%)	142 (56.57%)	139 (55.38%)	0.8454
	II	52 (10.36%)	28 (11.16%)	24 (9.56%)	
	III	112 (22.31%)	53 (21.12%)	59 (23.51%)	
	IV	55 (10.96%)	26 (10.36%)	29 (11.55%)	
	unknown	2 (0.4%)	2 (0.8%)	0 (0%)	
T	T1	143 (28.49%)	68 (27.09%)	75 (29.88%)	0.6897
	T2	164 (32.67%)	88 (35.06%)	76 (30.28%)	
	T3	170 (33.86%)	82 (32.67%)	88 (35.06%)	
	T4	23 (4.58%)	11 (4.38%)	12 (4.78%)	
	unknown	2 (0.4%)	2 (0.8%)	0 (0%)	
M	M0	282 (56.18%)	143 (56.97%)	139 (55.38%)	0.0555
	M1	9 (1.79%)	1 (0.4%)	8 (3.19%)	
	unknown	211 (42.03%)	107 (42.63%)	104 (41.43%)	
N	N0	229 (45.62%)	114 (45.42%)	115 (45.82%)	0.7029
	N1	223 (44.42%)	116 (46.22%)	107 (42.63%)	
	unknown	50 (9.96%)	21 (8.37%)	29 (11.55%)	

### Establishment and validation of prognostic model

**FIGURE 4:**
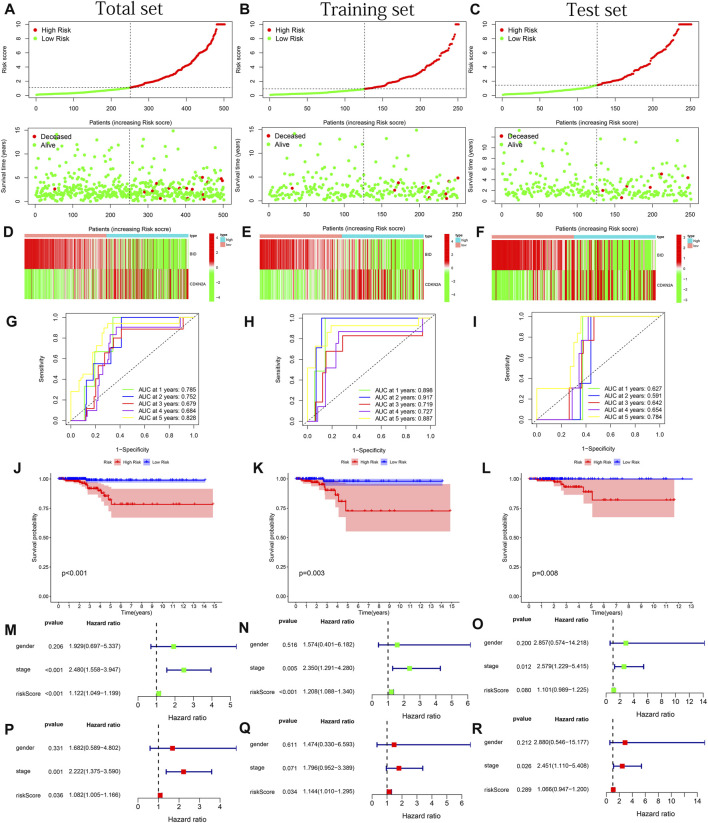
Model performance test based on the total, training and test sets. **(A–C)** Risk curves and survival status graphs. **(D–F)** Expression heatmap of 2 PR-DE-IRFeGs. **(G–I)** ROC curves for 1–5 years. **(J–L)** Kaplan-Meier survival curves. **(M–O)** Forest plots of univariate Cox regression. **(P–R)** Forest plots of multivariate Cox regression.

CDKN2A and BID were determined based on the finest λ value by lasso regression analysis and used to construct a multivariate Cox regression model (Supplementary Figures S3A–B). And the co-expression relationship between these PR-DE-IRFeGs and the corresponding DE-IRGs was shown in Figure 3E. CDKN2A’s low expression and BID’s high expression are associated with better OS (Figures 3F,G). Table 1 clearly shows that randomization did not result in significant differences of the clinical characteristics between different sets. We calculated the risk score of each sample in the training, test and total sets from TCGA using the risk score calculation formula: risk score = (CDKN2A expression value *−1.6634 + BID expression value *1.2448). Following that, all samples were computed based on their sets. The sample’s risk score was compared to the set’s median risk score, and the samples were divided into high-risk and low-risk groups. Figures 4A–C shows the risk profile and survival status plots that show the distribution of risk scores, as well as the OS of each sample in 3 sets. The heatmap also displays the various risk scores, as well as the various expression levels of the two PR-DE-IRFeGs (Figures 4D–F). Most of the AUCs for the 3 sets were above 0.65, which was satisfactory (Figures 4G–I). The Kaplan-Meier curve shows that the survival probability of patients in the high-risk group is lower than that of patients in the low-risk group (Figures 4J–L), indicating that the high-risk group’s prognosis is poor. As a result, independent of interference from clinical factors (gender and clinical stage), the risk score was an independent predictor of OS in both the total and training sets (Figures 4M–Q). Unfortunately, this outcome did not show up in the test set in a meaningful way (Figures 4O–R). In conclusion, our model performs well in predicting prognosis of patients with THCA.

### Comparison of the performance of various prognostic models

**FIGURE 5:**
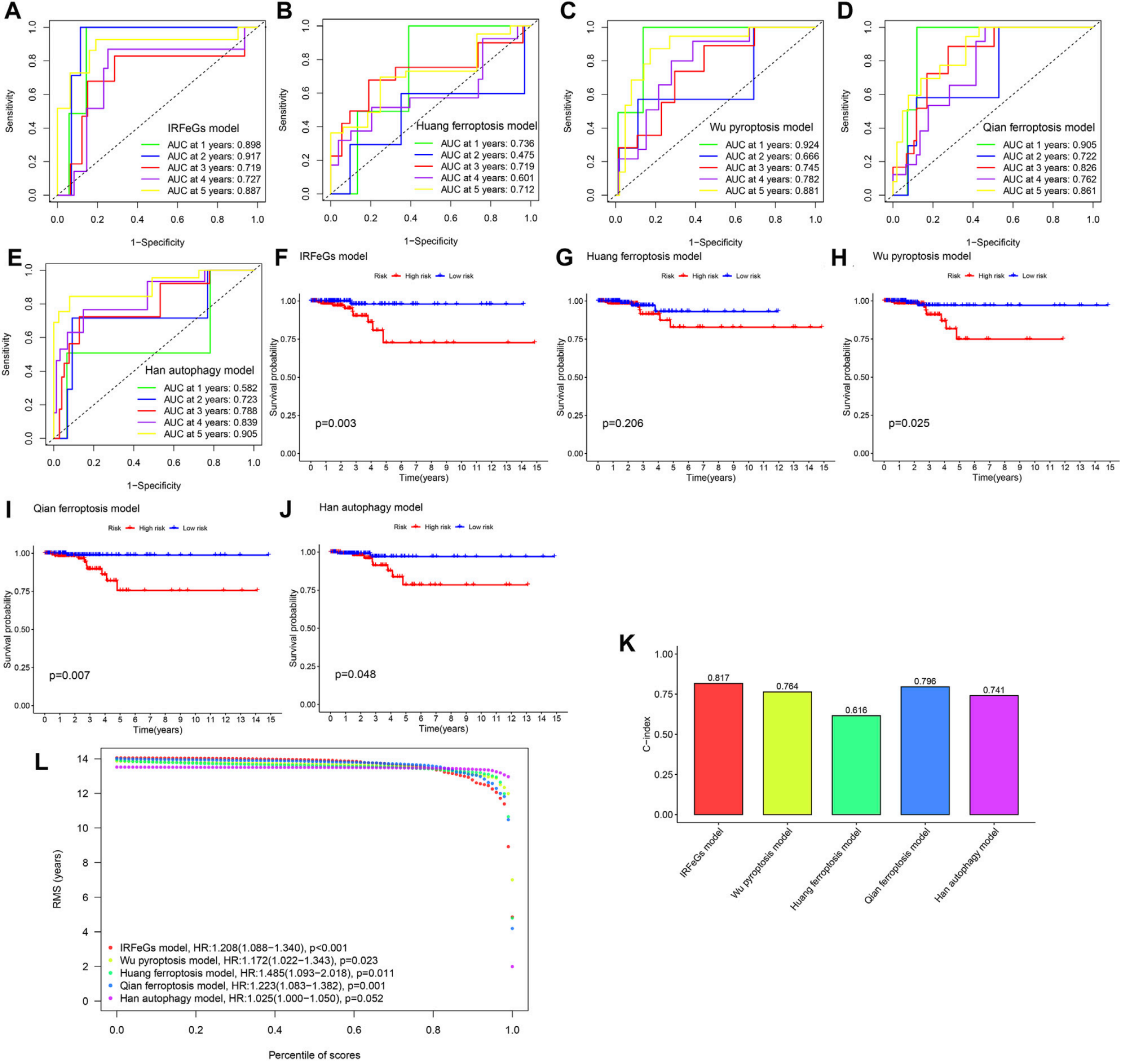
Performance comparison among different models. **(A–E)** The 1–5 years ROC curves of 5 models, respectively. **(F–J)** Kaplan-Meier survival curves of 5 models, respectively. **(K)** Comparison of C-index among the 5 models. **(L)** Comparison of RMST curves among the 5 models.

When the ROC curves were compared, our model had the most outstanding AUC value in all years (Figures 5A–E), implying the most prominent performance of our model. Our model has a greater capacity to identify prognosis than other models, as shown by the Kaplan-Meier survival curves (Figures 5F–J). Not only that, but by comparing the c-indexes, we discovered that our model outperformed all others (Figure 5K). In addition, our model was observed to have the highest RMST curve out of five (Figure 5L).

### 2 DE-PR-IRFeGs protein levels’ differential expression

**FIGURE 6:**
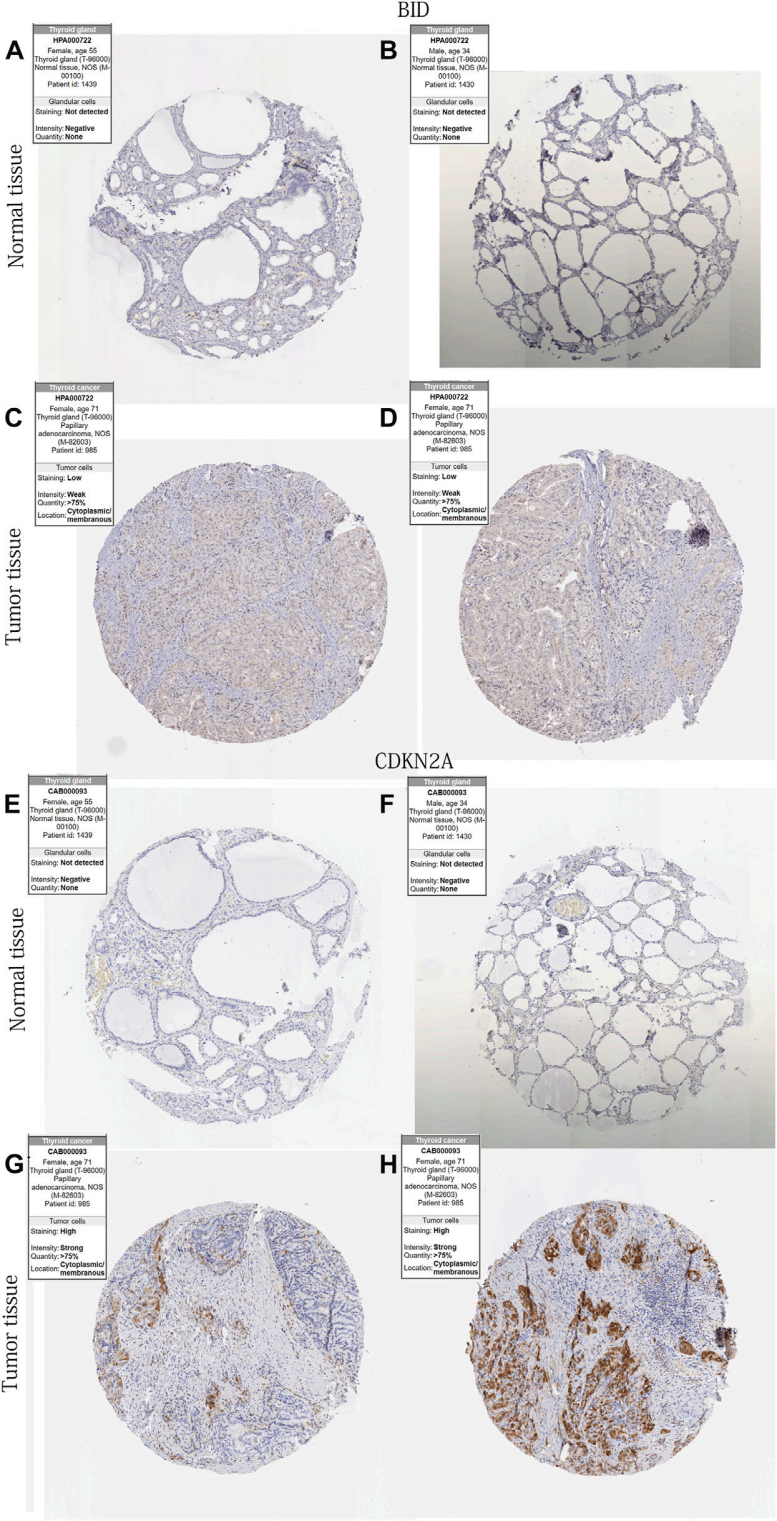
2 DE-PR-IRFeGs protein levels’ differential expression in protein level. **(A–H)** BID and CDKN2A protein expression level in normal thyroid and THCA tissues. Kaplan-Meier survival curves in different subgroups of each clinical feature.

Figures 6A–H showed IHC staining of CDKN2A and BID protein expression in THCA and normal thyroid tissue. The results showed higher expression of both genes in THCA tissues.

### Assessment of our model performance around clinical subgroups.

We used the Kaplan-Meier survival curve to test the model’s predictive performance in various clinical subgroups (Supplementary Figures S4A–L). Except for the age ≤60 years, stage I-II, and M1 subgroups (*p* > 0.05), our model maintained strong performance in identifying OS in most subgroups (p 0.05). Patients in all subgroups with a high risk have a terrible prognosis.

### GSEA enrichment analysis of high-risk and low-risk groups

Supplementary Figures S5A,B showed the results of biological functions enrichment based on high-risk and low-risk groups, respectively. The GO-enriched biological functions in the high-risk group mainly include fatty acid metabolic process, oxidative phosphorylation, skeletal system development, and thyroid hormone generation. Moreover, the low-risk group mainly includes interferon-gamma production, response to type I interferon, T-cell activation involved in immune response, T-cell mediated immunity, T-helper1-type immune response, type-I interferon production. The KEGG pathway in the low-risk group was mainly enriched in: cell adhesion junction, cell adhesion molecules cams, cytokine-cytokine receptor interaction, natural killer cell-mediated cytotoxicity (Supplementary Figure S6C). The main enriched KEGG pathways in the high-risk group are arginine and proline metabolism, butanoate metabolism, glycine serine, threonine metabolism, oxidative phosphorylation, and propanoate metabolism (Supplementary Figure S6D). The enrichment results for these risk groups were all strongly associated with immunity and ferroptosis.

### Immune cell and immune function analysis

**FIGURE 7:**
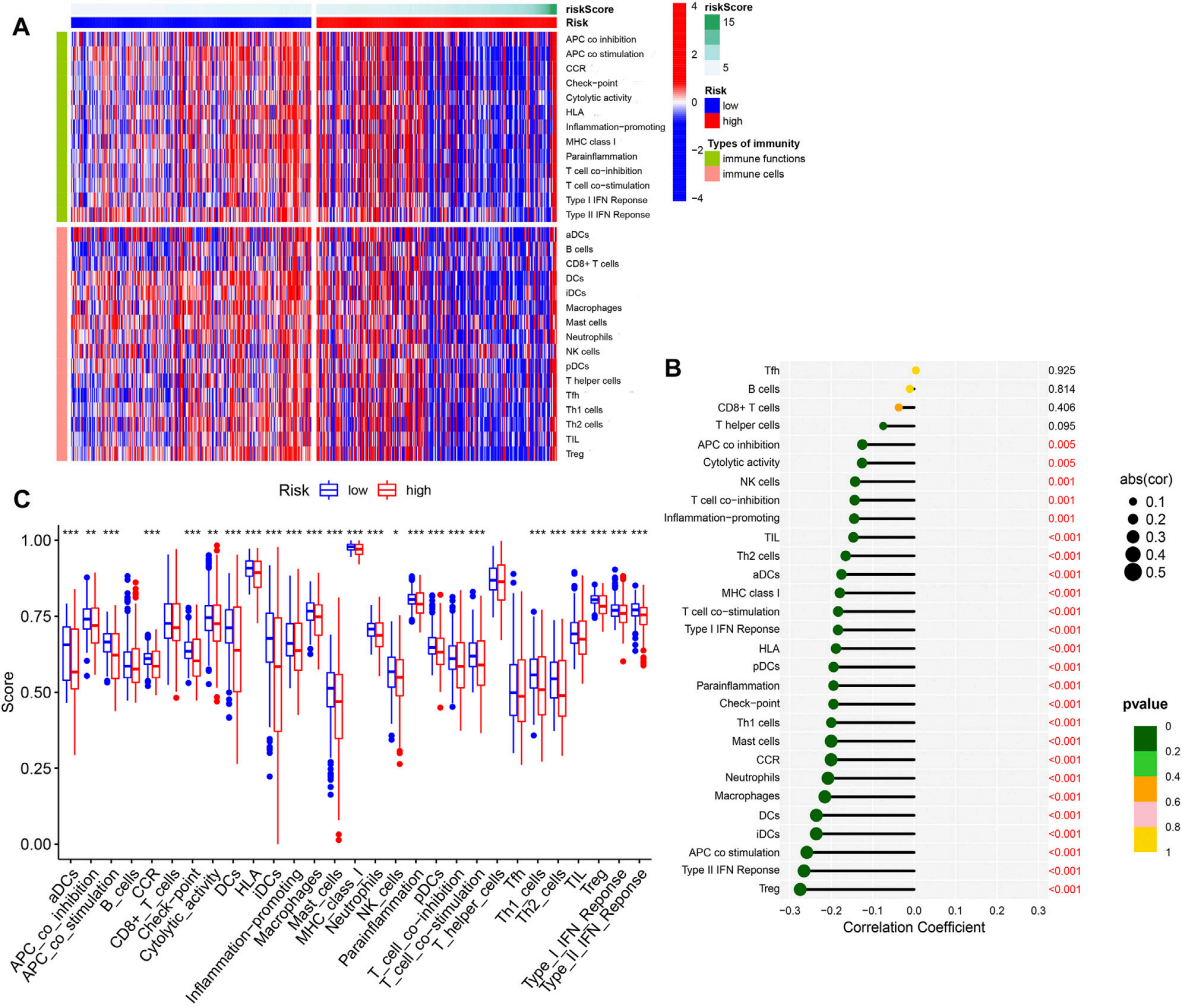
Immune infiltration analysis. **(A)** Heat map of the distribution difference of 16 kinds of immune cells and 13 kinds of immune functions. **(B)** The correlation between 16 types of immune cells/13 types of immune functions and risk scores. **(C)** Difference analysis of 16 immune cells and 13 immune functions in different risk groups.

The 2 DE-PR-IRFeGs of the model were closely related to immune genes and ferroptosis genes, and the results of GSEA analysis also verified the correlation between our model and immunity/ferroptosis. Figure 7A showed the distribution of immune cell/immune function scores for each sample as the risk score increased. As shown in Figure 7B, except for T follicular helper cells (TFH), B cells, CD8 + T cells, and T helper cells, the negative correlation between other immune cell/function scores and risk scores is statistically significant. Figure 7C supported this result with difference analysis of these scores between different risk groups. In conclusion, we can determine the close relationship between the risk score and most immune cells/immune functions.

### Prediction of a network regulatory network targeting CDKN2A

**FIGURE 8:**
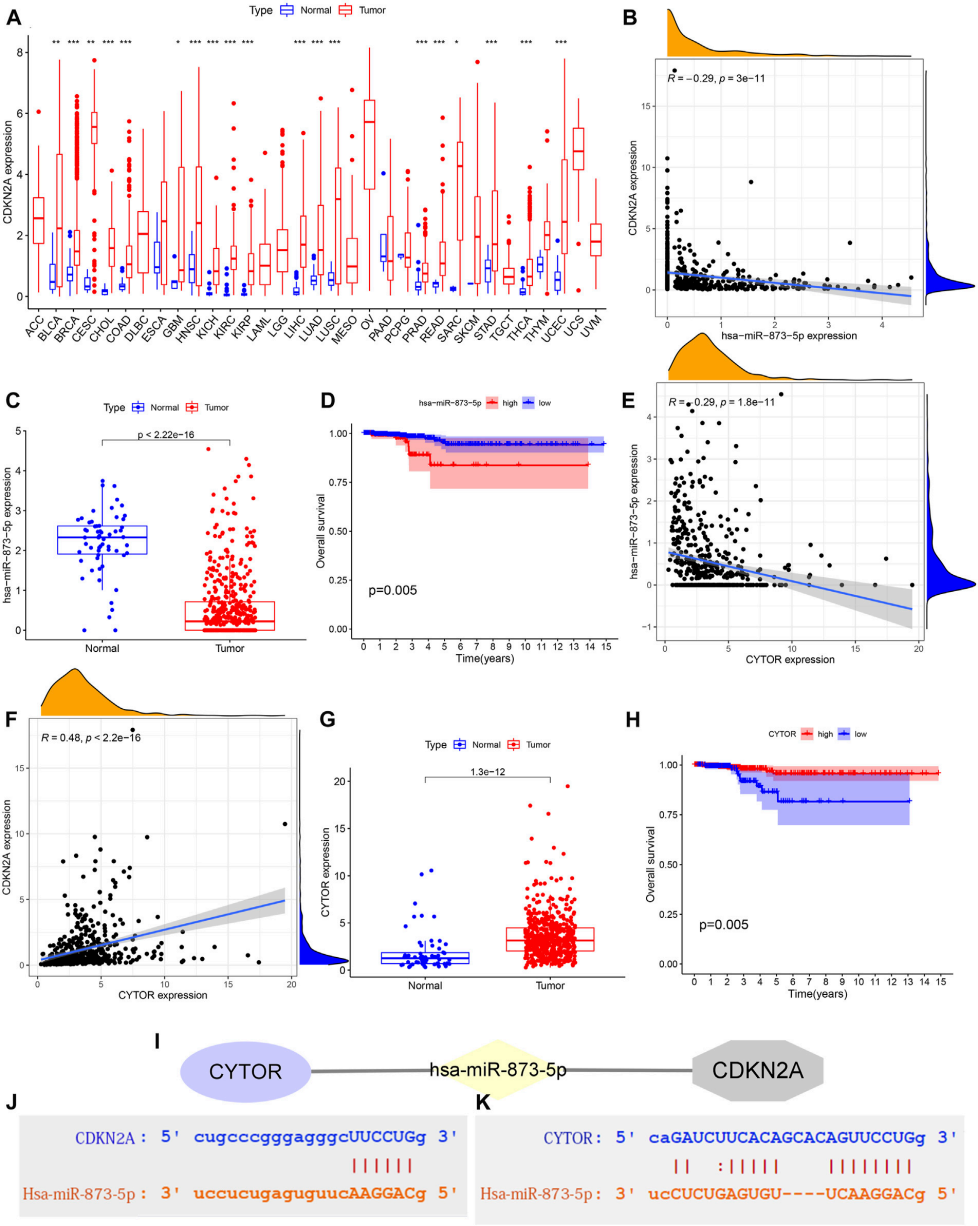
Prediction of a network regulatory network targeting CDKN2A. **(A)** CDKN2A expression difference between 33 cancers and normal tissues. no logo: not significant; *: *p* < 0.05; **: *p* < 0.01; ***: *p* < 0.001. **(B)** Correlation analysis between CDKN2A and hsa-miR-873-5p expression. **(C)** The expression difference of hsa-miR-873-5p between normal and THCA tissues. **(D)** Survival probability difference between high and low expressed hsa-miR-873-5p. **(E)** Correlation analysis of CYTOR and hsa-miR-873-5p expression. **(F)** Correlation analysis of CYTOR and CDKN2A expression. **(G)** Differences in CYTOR expression between normal and THCA tissues. **(H)** Differences in survival probability between high and low CYTOR expression groups. **(I)** CeRNA regulatory network composed of CYTOR, hsa-miR-873-5p and CDKN2A. **(J)** The predicted binding sites of hsa-miR-873-5p and CDKN2A. **(K)** The predicted binding sites of hsa-miR-873-5p and CYTOR.

There are significant differences in the expression of CDKN2A in many cancers (Figure 8A). Moreover, CDKN2A was up-regulated in these cancers. There are 84 upstream miRNAs that we predicted that may bind to CDKN2A. Hsa-miRNA-873-5p has a significant negative correlation with CDKN2A (Figure 8B). Hsa-miRNA-873-5p was significantly down-regulated in THCA (Figure 8C), and THCA patients with low expression of hsa-miRNA-873-5p showed a better prognosis (Figure 8D). All the above results indicated that hsa-miRNA-873-5p is a miRNA with significant biological value in THCA that may regulate the expression of CDKN2A. Among the 108 lncRNA obtained from the StarBase database, only CYTOR met the statistical threshold condition. CYTOR expression was negatively correlated with hsa-miRNA-873-5p expression and positively correlated with CDKN2A expression (Figures 8E,F). Relative to normal tissues, CYTOR was more expressed in tumor tissues, as shown in Figure 8G. Low CYTOR expression, on the other hand, was linked to a poor THCA patients’ prognosis (Figure 8H). Figure 8I depicted the ceRNA regulation network, including CYTOR, hsa-miRNA-873-5p and CDKN2A. Figures 8J,K showed the predicted binding sites of hsa-miR-873-5p and CDKN2A as well as hsa-miR-873-5p and CYTOR obtained from the Starbase website.

### Model closely links to gene mutations

**FIGURE 9:**
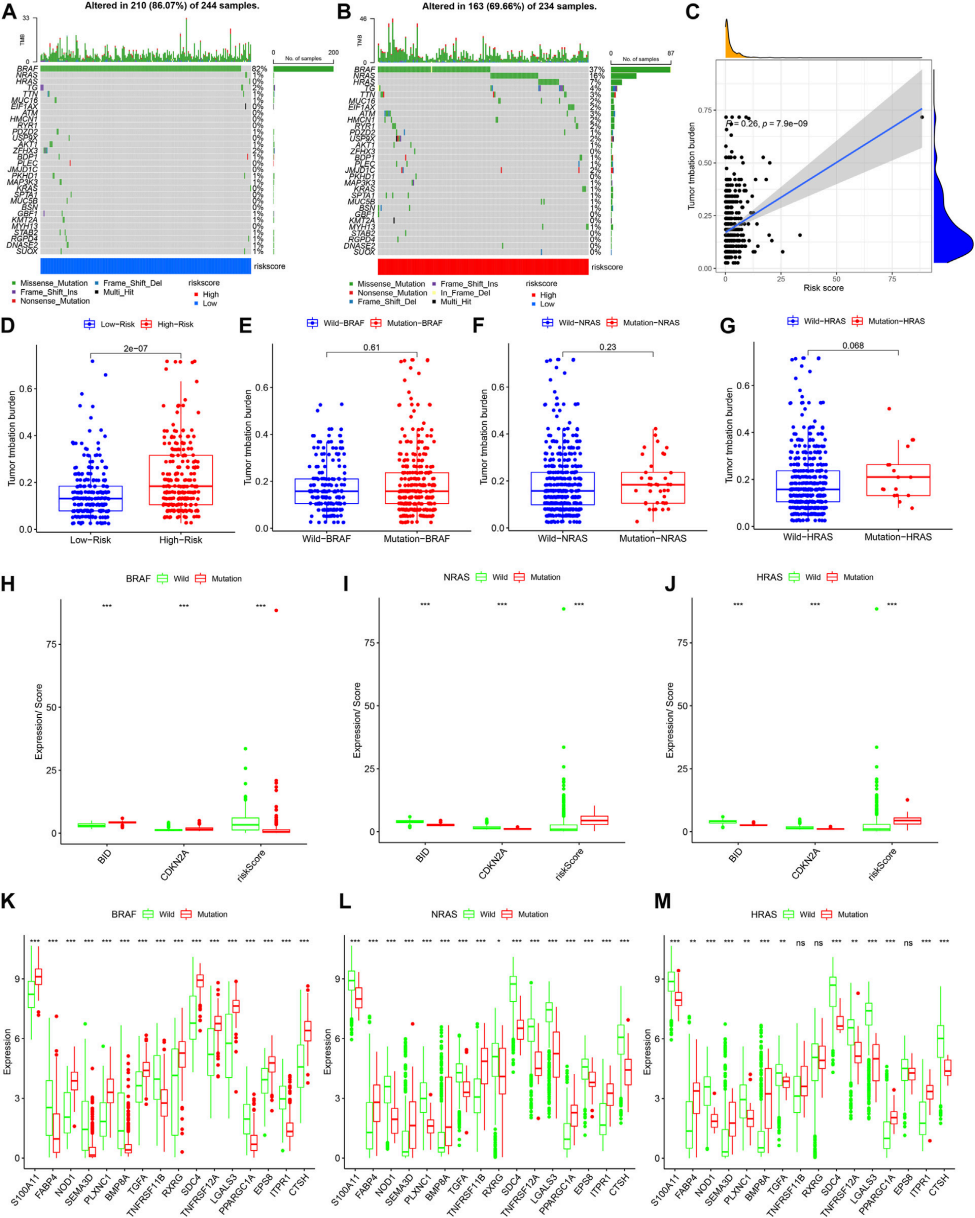
Model closely links to gene mutations. **(A,B)** Waterfall charts show mutations in the top 30 most common genes in different risk groups, respectively. The right panel of the waterfall plot shows mutation frequencies, and the different colors at the bottom of the graph show subgroups of different mutation types and clinical features. The histogram above is the TMB statistic result for each sample. **(C)** Correlation between TMB and risk score. **(D)** TMB differences in between high and low risk groups. **(E–G)** TMB differences between BRAF/NRAS/HRAS wild and mutant groups. **(H–J)** Differences in the BID, CDKN2A expression and risk score between BRAF/NRAS/HRAS wild and mutant groups. **(K–M)** Differences in the expression of DE-IRGs upstream of BID and CDKN2A between the BRAF/NRAS/HRAS wild and mutant groups, respectively. ns: not significant; *: *p* < 0.05; **: *p* < 0.01; ***: *p* < 0.001.

After sorting the gene mutation frequencies across all TCGA samples, we visualized the top 30 gene mutations in the low-risk and high-risk groups (Figures 9A,B). As can be seen from the figure, BRAF, NRAS, and HRAS have the highest mutation frequency. And the high-risk group had higher TMB (Figure 9D). The correlation analysis between the risk score and TMB in Figure 9C further confirmed the positive correlation between the risk score and TMB. Unfortunately, no significant differences were found for TMB between the BRAF/NRAS/HRAS wild and mutant groups (Figures 9E–G). BRAF mutations were associated with higher BID, CDKN2A expression and lower risk scores (Figure 9H). Low BID, CDKN2A expression, and higher risk scores were found in the NRAS and HRAS mutation groups (Figures 9I,J). Figure9K shows the differences in PR-DE-IRGs associated with BID and CDKN2A between the BRAF mutation and the wild groups. Interestingly, the corresponding results for NRAS and HRAS were opposite to those for BRAF (Figures 9L,M).

### CNV analysis

**FIGURE 10:**
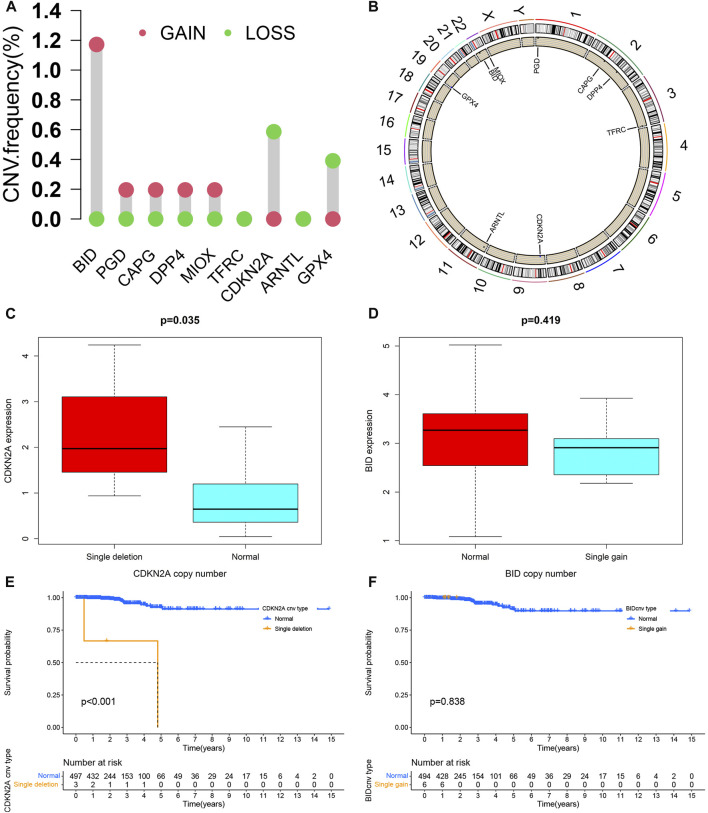
CNV analysis. **(A)** The statistics result of CNV of 9 PR-DE-IRFeGs. The green dot represents the loss of CNV, while the pink dot represents the gain of CNV. **(B)** The position distribution of CNV changes of 9 PR-DE-IRFeGs on 23 chromosomes. **(C)** Differences in CDKN2A expression between CDKN2A single deletion and normal groups. **(D)** Differences of BID expression in BID single gain copy number and normal group. **(E,F)** Kaplan-Meier survival curves between different copy number variation groups of BID/CDKN2A, respectively.

The CNVs of 9 PR-DE-IRFeGs we found were shown in Figure 10A. BID, PGD, CAPG, DPP4, and MIOX have higher CNV gain frequencies, while CDKN2A and GPX4 have higher CNV loss frequencies. The chromosomal locations of these genes were shown in Figure 10B. These genes are mainly located on chromosomes 1, 2, 3, 9, 11, 19, and 22. Higher CDKN2A expression was associated with its single deletion copy number (Figure 10C). Unfortunately, we did not observe a significant relationship between BID expression and its single gain copy number (Figure 10D). Likewise, CDKN2A single deletion copy number group had a poorer survival probability (Figure 10E). However, we did not find a clear relationship between BID single gain copy number and survival probability (Figure 10F).

### Treatment Predictive Efficacy of the model

**FIGURE 11:**
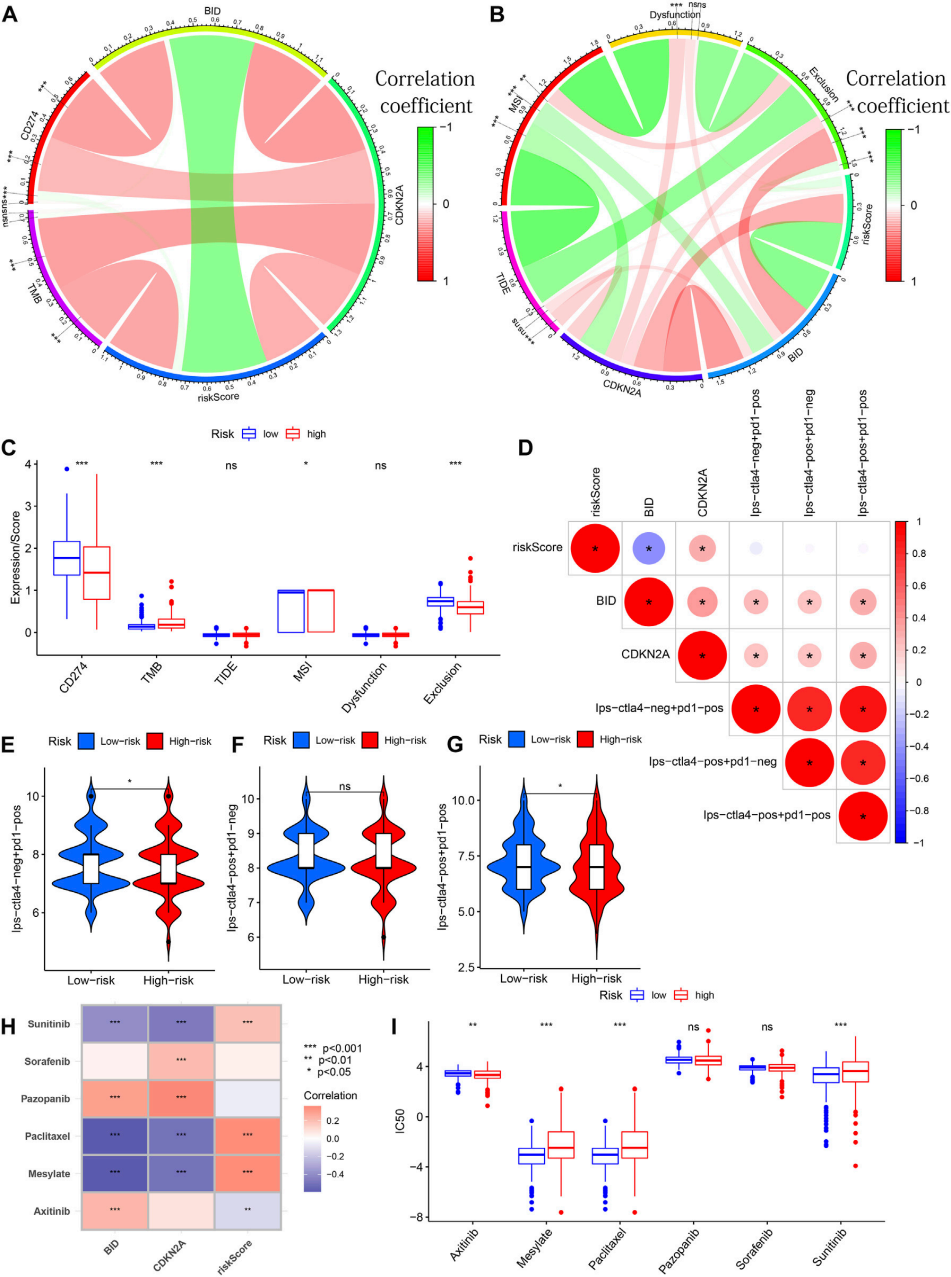
Treatment Predictive Efficacy of the Model. **(A)** Correlation analysis between BID/CDKN2A expression/risk score and CD274/TMB. **(B)** Correlation analysis between the BID/CDKN2A expression/risk scores and TIDE/MSI/dysfunction/exclusion scores. **(C)** Differences between CD274, TMB, TIDE, MSI dysfunction and exclusion scores between different risk groups. **(D)** Correlation of 3 IPSs with risk score/BID/CDKN2A expression. **(E–G)** Differences of 3 IPSs between different risk subgroups. **(H)** Correlation between IC50 of 6 chemotherapy drugs and risk score/BID/CDKN2A expression. **(I)** Differential analysis of 6 chemotherapeutic drugs in different risk groups. In circular or lollipop charts: ns: not significant; *: *p* < 0.05; **: *p* < 0.01; ***: *p* < 0.001.

From Figure 11A, we can see that CDKN2A expression is positively correlated with CD274 expression and TMB. A positive correlation between BID and CD274 expression as well as a positive correlation between risk score and TMB was observed. The CNKN2A was positively correlated with TIDE (Figure 11B). There were differences in CD274 expression, TMB, MSI and exclusion between the high and low-risk groups (Figure 11C). Meanwhile, these 2 genes were found to be positively associated with 3 IPSs (Figure 11D). There were differences in Ips-ctla4-neg + pd1-pos and Ips-ctla4-pos + pd1-pos, while there was no significant difference in Ips-ctla4-pos + pd1-neg between different risk groups (Figures 11E–G). We found that Sunitinib, Paclitaxel and Mesylate were significantly positively correlated with risk scores (Figure 11H). The correlation study was further supported by the difference in IC50 of the three medicines between the high and low-risk groups (Figure 11I). Significant correlations between 2 PR-DE-IRFeGs expression and IC50 of some drugs were also observed. The above results all illustrate the predictive efficacy of our model and the genes within the model.

### Combination of a nomogram for predicting patients’ survival probability

**FIGURE 12:**
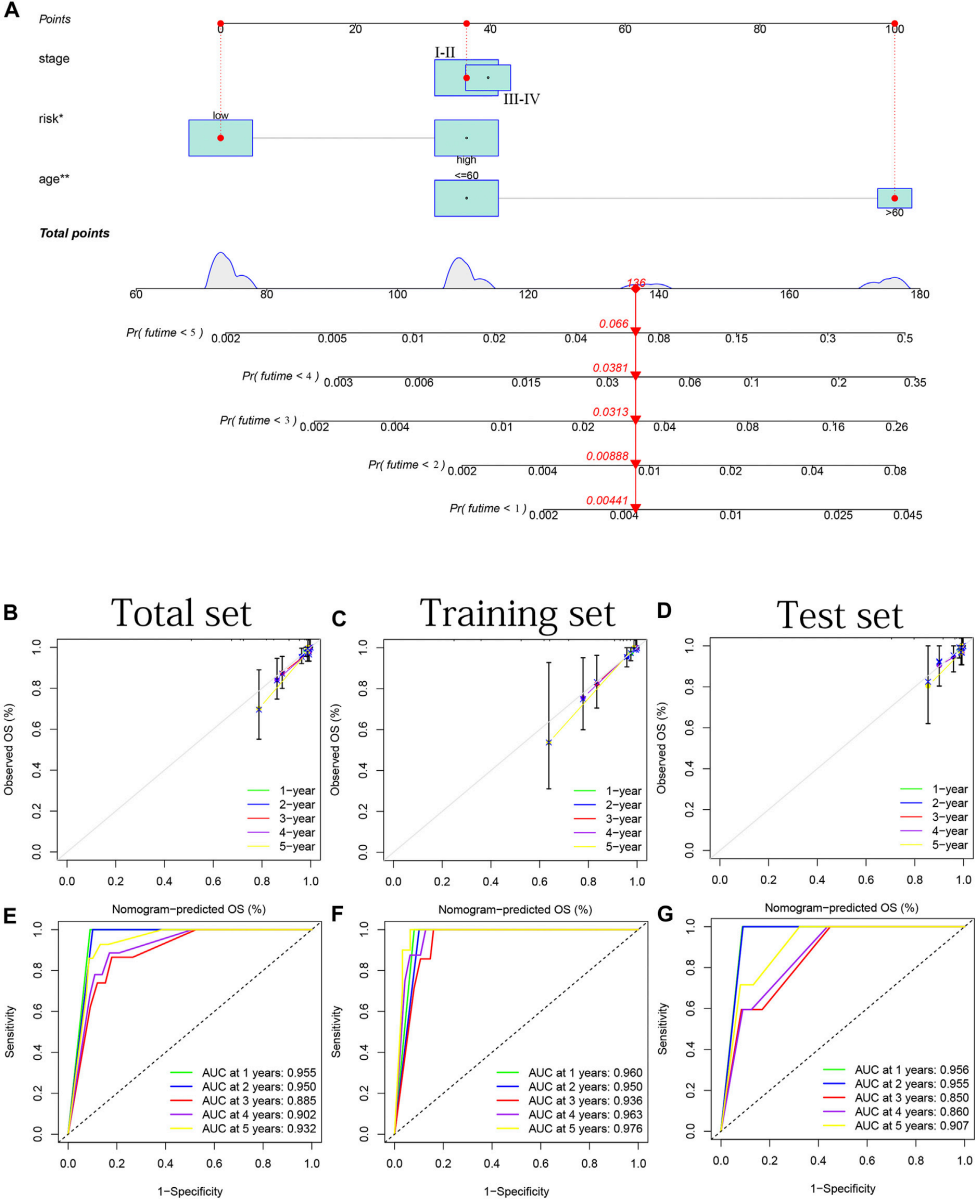
Combination of a nomogram for predicting patients’ survival probability. **(A)** Nomogram based on two clinical criteria (age and stage) and risk group. **(B–D)** 1–5 years’ internal calibration curves based on the total, training and test sets, respectively. **(E–G)** 1–5 years’ ROC curves based on the total, training and test sets, respectively.

A nomogram based on two clinical criteria (age and stage) as well as the risk group was shown in Figure 12A. In Figures 12B–D, we discovered parallels between the predicted and actual OS in all years based on three sets data. These results suggest that our nomogram can serve as an accurate tool for predicting survival probability in THCA patients. In Figures 12E–G, we can observe that the time-dependent ROC curve in all years (most AUC >0.85) indicated the nomogram’s good predictive capacity.

### Validation of the differential expression of 2 PR-IRFeGs using an external cohort

**FIGURE 13:**
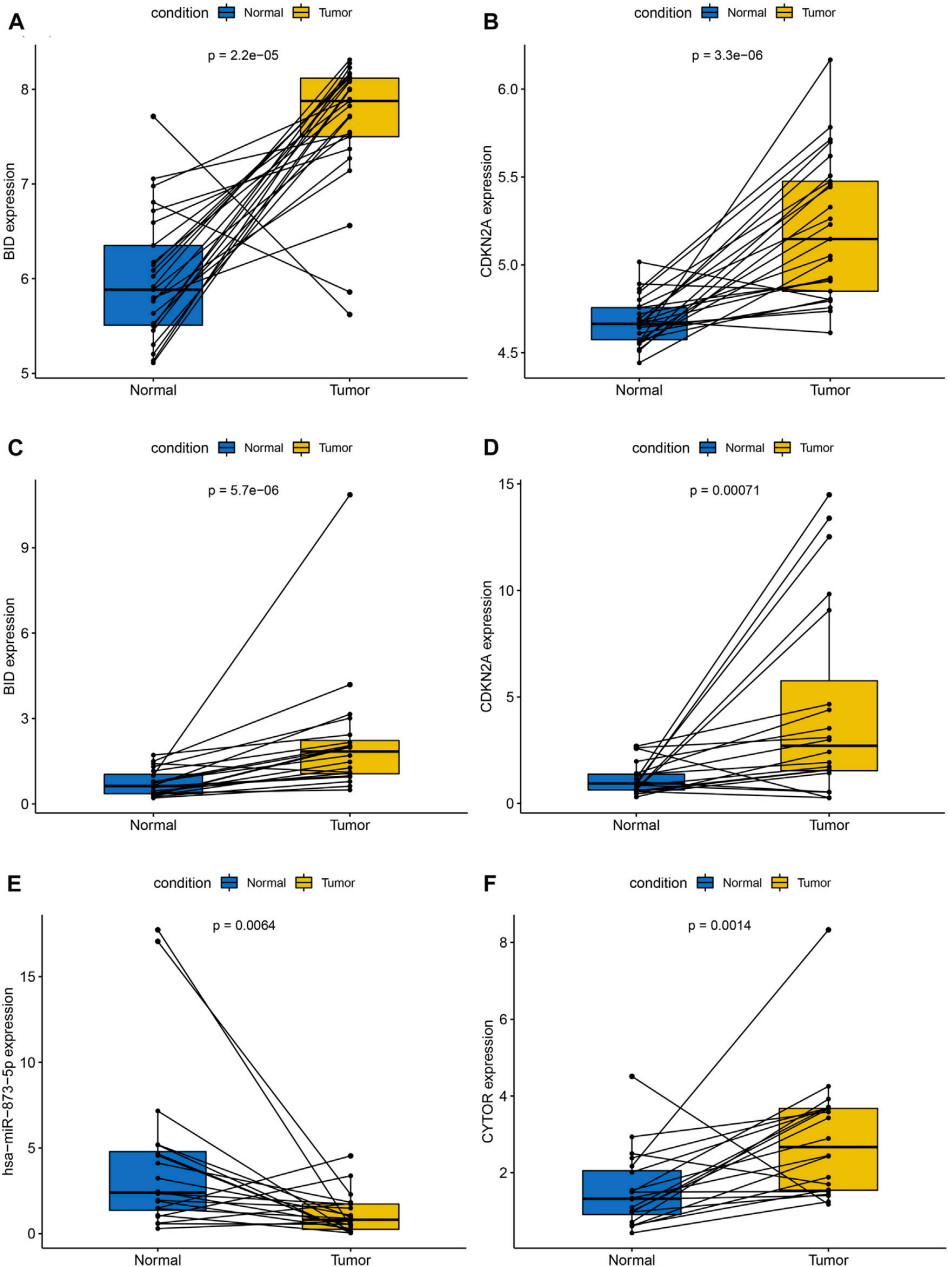
External cohort validation of 2 PR-IRFeGs and pairwise comparison of relative expression of 4 genes detected by qRT-PCR. External cohort validation: **(A)** BID. **(B)** CDKN2A. qRT-PCR: **(C)** BID. **(D)** CDKN2A. **(E)** hsa-miR-873-5p. **(F)** CYTOR.

In the GSE60542 external cohort, we observed higher expression of 2 PR-IRFeGs in THCA (Figures 13A,B), which is consistent with the analysis results of the TCGA, GSE33630 and GSE35570 cohorts.

### Using qRT-PCR to verify the relative expression differences of related genes

Paired boxplots showed significant differences in the relative expression of all 4 genes between THCA tissue and adjacent normal thyroid tissue (Figures 13C–F). We observed higher expression of BID, CDKN2A and CYTOR as well as lower expression of hsa-miR-873-5p in THCA tissue compared with adjacent normal thyroid tissue, all of which were consistent with our previous results.

## Discussion

Our study extracted 35 PR-DE-IRGs and 10 PR-DE-FRGs from TCGA and GEO databases for co-expression analysis. CDKN2A and BID were identified as prognostic risk and protective factors, respectively. External data and qRT-PCR experiment also validated their differential expression. They were finally screened to construct a prognostic model. The model’s excellent performance has been repeatedly verified and outperformed other models. The model and composite nomogram also demonstrated excellent clinical value. This is reflected in the close correlation between BID/CDKN2A/risk score and TIDE/CD274 expression/TMB/IPS/IC50 of 6 chemotherapeutic drugs. The multi-perspective multi-omics analysis also provided many valuable results. The significant correlation of risk score with most immune cells/function and the close correlation of risk score/2 PR-DE-IRFeGs expression with BRAF/NRAS/HRAS mutation provided valid evidence for further mechanistic exploration. The close association of single-copy number deletion of CDKN2A with upregulation of CDKN2A expression/poor prognosis and the CYTOR-hsa-miRNA-873-5p-CDKN2A regulatory network that significantly affects prognosis both support their potential roles in THCA progression.

BID, a member of the Bcl-2 family, has a pro-apoptotic function (Wang et al., 1996; Esposti, 2002; Billen et al., 2008; Gahl et al., 2016), and is closely related to the induction of ferroptosis and mitochondrial damage (Neitemeier et al., 2017; Jelinek et al., 2018; Wang et al., 2021b; Li et al., 2021). Moreover, BID also plays an inhibitory role in gastric cancer, ovarian cancer, pancreatic cancer and other cancers (Sinicrope et al., 2008; Goncharenko-Khaider et al., 2010; Aranovich et al., 2012; Gryko et al., 2014). These results, like our study, suggest that BID can be a protective factor in cancers. CDKN2A on chromosome 9p21 encodes p16INK4a and p14ARF proteins, whose mutation or deletion are closely related to various tumors, such as pancreatic cancer, thymic cancer, melanoma, lung cancer, and others (Sherborne et al., 2010; Nikolaou et al., 2011; Aesif et al., 2017; Cancer Genome Atlas Research Network, 2017; Satpathy et al., 2021; Singhi and Wood, 2021). These p16INK4a overexpressed tumors are usually highly invasive (Romagosa et al., 2011). Overexpression of p14ARF and p16INK4a has also been found in invasive areas of head and neck squamous cell carcinoma (Natarajan et al., 2003), colorectal carcinoma (Jung et al., 2001) and endometrial carcinoma (Horrée et al., 2007). These results help to demonstrate that CDKN2A may act as a risk factor in cancers. It has been reported that overexpression of p14ARF and p16INK4a was observed in follicular adenoma, follicular carcinoma, and papillary carcinoma, which is consistent with our observation (Ferru et al., 2006). The reason may be that excessive abnormal cell growth triggers CDKN2A expression.

We found that the high-risk group is mainly enriched in fatty acid metabolism and oxidative phosphorylation because tumor cell proliferation requires fatty acid synthesis to synthesize cell membranes and signaling molecules (Carracedo et al., 2013; Currie et al., 2013; Röhrig and Schulze, 2016). A large amount of evidence shows that even if there is active glycolysis in tumors, the role of oxidative phosphorylation in tumors cannot be ignored (Moreno-Sánchez et al., 2007; Weinberg and Chandel, 2015; Ashton et al., 2018). The enrichment pathway in our low-risk group is mainly related to immune response. The antitumor immune response inhibits tumor growth, and the active immune response is key to improving survival results (Yamaguchi and Sakaguchi, 2006; Gajewski et al., 2013; Li et al., 2016; Savas et al., 2016). Surprisingly, further analysis also found that most immune cells were more abundant in the low-risk group.

In recent years, ferroptosis induction has emerged as an alternative and/or combination therapy approach to trigger cancer cell death, especially for the treatment of malignancy resistance issues in certain cancers (Xu et al., 2019; Lin et al., 2021). At present, there are a large number of reports indicating that ferroptosis is closely related to immune cells and immune responses (Levring et al., 2012). Macrophages can generate oxygen radicals through the membrane-associated NADPH oxidase complex or through the mitochondrial electron transport chain, and ferroptosis requires the use of iron-dependent oxygen radicals for membrane lipid peroxidation (Vazquez-Torres et al., 2000; Dixon et al., 2012). IFN-γ released by CD8^+^ T cells downregulates the expression of two subunits of the glutamate-cysteine anti-transport system, SLC3A2, and SLC7A11, inhibits the uptake of cystine by tumor cells and promotes tumor cells lipid peroxidation and ferroptosis (Yamazaki et al., 2014; Wang et al., 2019). This indicates that cancer cells undergoing ferroptosis release HMGB1, an important protein necessary to make cancer cells immunogenic, and DAMPs, an endogenous molecule released from injured or stressed cells (Tang et al., 2012; Yu et al., 2015; Wen et al., 2019; Zindel and Kubes, 2020; Chen et al., 2021). DAMPs bind to pattern recognition receptors (PRRS) after dendritic cell recruitment, dendritic cell-mediated antigen capture, and presentation. PRRS signals activation of downstream transcription factors such as NF- κ B and interferon regulatory factor (IRF) and produces a variety of immune factors such as cytokines and chemokines, thereby inducing cytotoxic T cell responses (Chen et al., 2021). DAMPs-PRR axis plays a central role in bridging cell death and immune response in tumor immunity (Tang et al., 2012; Zindel and Kubes, 2020). Wang et al. found that lipid peroxidation caused by ferroptosis activator elastin promoted the proliferation and differentiation of human peripheral blood monocytes into natural killer cells and B cells by down-regulating bone morphogenetic protein (BMP) (Wang et al., 2018). It shows that ferroptosis also contributes to the immune response of tumor cells. By analyzing the above evidence, it can be speculated that the low-risk group has a good prognosis and is enriched with more immune-related collaterals, which may be closely related to the occurrence of ferroptosis and immune function.

As a general term for a class of RNAs, including mRNAs, lncRNAs, circRNAs, etc., ceRNAs have miRNA binding sites that can compete with miRNAs to inhibit the regulation of target genes (Das et al., 2014; Tay et al., 2014). With numerous in-depth research, the changes of target gene expression caused by ceRNA regulatory network have become more and more prominent in cell metabolism and the occurrence and development of cancer (Karreth and Pandolfi, 2013). From the results of our analysis, it can be seen that CDKN2A is significantly overexpressed in THCA and exhibits a significant effect on prognosis. Currently, there are few ceRNA studies targeting CDKN2A in THCA. Therefore, it is very necessary to further predict the ceRNA network potentially regulating CDKN2A expression. In this study, we predicted that the upstream regulators of CDKN2A were hsa-miRNA-873-5p, which was significantly down-regulated in THCA (Zou et al., 2021). In addition to THCA, hsa-miRNA-873-5p is also down-regulated in as many as 13 other cancers, such as lung cancer (Jin et al., 2018). It has been shown to play an oncogenic role in lung adenocarcinoma cells (Gao et al., 2015) and hepatocellular carcinoma (Li et al., 2018; Zhang et al., 2019). This is consistent with the Kaplan-Meier survival curve results for THCA in our study. But a previous review demonstrated that miR-873-5p may promote or inhibit cancer progression, which suggests that the effect of miR-873-5p on cancers remain controversial. At the same time, we found that the lncRNAs with the strongest correlation with hsa-miRNA-873-5p was CYTOR, which was highly expressed in THCA, and suggested a better prognosis. Studies have shown that CYTOR can regulate gene expression through various mechanisms as a significant oncogene (Neumann et al., 2012; Chen et al., 2016; Yue et al., 2016). However, there are still studies reporting its knockdown on the promotion of glioma progression, which is consistent with our results (Binder et al., 2021). Furthermore, the effects of miR-873-5p and CYTOR on THCA were divergent from those produced by other tumors identified in the literature review, possibly due to the prominent tumor heterogeneity of THCA (Binder et al., 2021). CYTOR is also up-regulated in colon cancer (Yue et al., 2016) and gastric cancer (Chen et al., 2016). CYTOR and hsa-miRNA-873-5p are negatively correlated in THCA (Wu et al., 2020). The above results implied that CYTOR and hsa-miRNA-873-5p, as key genes of potential regulatory network, could affect the THCA’s progression and prognosis of by regulating CDKN2A expression.

TMB’s higher-level reflects lower survival in many cancers (Eder et al., 2019; Hwang et al., 2019; Wu et al., 2019; Doig et al., 2022), which is consistent with our findings: the model risk score was positively associated with the occurrence of TMB. BRAF mutations are the most common mutations in papillary thyroid cancer (PTC) patients, occurring in up to 45% (Xing, 2005). Angell et al. (2014) observed that BRAF V600E in PTC was associated with increased PD1 ligand 1 expression by immunohistochemistry and direct DNA sequencing. Another study showed that patients with BRAF-mutated THCA could gain greater therapeutic values from combined BRAF and MEK inhibitor therapy (Cabanillas et al., 2018). In contrast, RAS mutation is the most common genetic alteration in poorly differentiated THCA, with an incidence of about 23% (Volante et al., 2009). The RAS mutation enhances the hypoxia-induced release of prototypic angiogenic factor (VEGF) in the microenvironment and promotes tumor progression (Shellman et al., 2003). This evidence may explain our observation that the BRAF mutant type had a lower risk score than the wild type, and the RAS mutant type had a higher risk score than the wild group. IPS can also reflect the tumor sensitivity to immunotherapy, similar to TMB. The 2 PR-DE-IRFeGs had positive correlations in different phenotypes, with higher IPS values in the low-risk group than high-risk group. It predicts that patients with low-risk scores will get more benefits from immunotherapy. We found that the IC50s of Sunitinib, Paclitaxel, and Mesylate were positively associated with our risk scores, also differing between high and low-risk groups. This suggests that our study is equally instructive for current targeted therapy and chemotherapy. Nomograph is an excellent method to evaluate the prognosis of tumors. The AUC value of the composite nomogram confirmed its excellent performance in predicting the survival rate of THCA patients.

As an important source of genetic variation, CNVs may lead to the heterogeneity of cancer genes, resulting in increased genetic instability, and are closely related to the development and prognosis of various cancers (Tanenbaum et al., 2016; Zhang et al., 2016). The deletion of the human CDKN2A gene frequently occurs in certain malignancies such as melanoma (Sun et al., 2004). In our study, CDKN2A single copy number deletion in THCA was associated with higher CDKN2A expression and poor prognosis. CDKN2A’s deletion has been previously reported to be associated with poor survival in anaplastic thyroid cancer or advanced differentiated thyroid cancers patients and poorest thyroid differentiation, which is consistent with our results (Yoshihara et al., 2013). Fang et al. also found that CDKN2A deletion is common in acute lymphoblastic leukemia, which is associated with poor prognosis (Fennell et al., 2022). A previous study has reported that RECQL4’s CNV are associated with its overexpression and increased breast cancer aggressiveness (Ashton et al., 2018). These all suggest that the single copy number deletion of CDKN2A may further promote the progression of THCA by upregulating the expression of CDKN2A.

However, our study has some limitations. First, the types of clinical data obtained from TCGA and GEO databases are limited, which prevents us from doing more in-depth analysis. Second, the two genes related to the prognosis model of PTC need further study on the molecular mechanism of tumors and the number and spatial morphological structure of translated proteins. Third, the nomogram has no detailed scores, including tumor size, tumor invasion and metastasis depth, tumor treatment, and surgical scope. In addition, some conclusions from this bioinformatics analysis, such as the close relationship between BRAF/NRAS/HRAS mutations and CDKN2A expression, still lack the support and scientific explanation based on basic experiments and valid literature reviews. The relevant conclusions do need to be confirmed in further research in the future. Finally, this study is a retrospective study, which needs to be verified by a more independent cohort and even a prospective clinical trial.

## Data Availability

The datasets presented in this study can be found in online repositories. The names of the repository/repositories and accession numbers can be found below: Gene Expression Omnibus, GSE33630 and GSE35570
