# Supplementary figures and images for: Identification of immune-related ferroptosis prognostic marker and in-depth bioinformatics exploration of multi-omics mechanisms in thyroid cancer
Source: Front Mol Biosci. 2022 Aug 17;9:961450. doi: 10.3389/fmolb.2022.961450 (PMC9428456; doi:10.3389/fmolb.2022.961450)

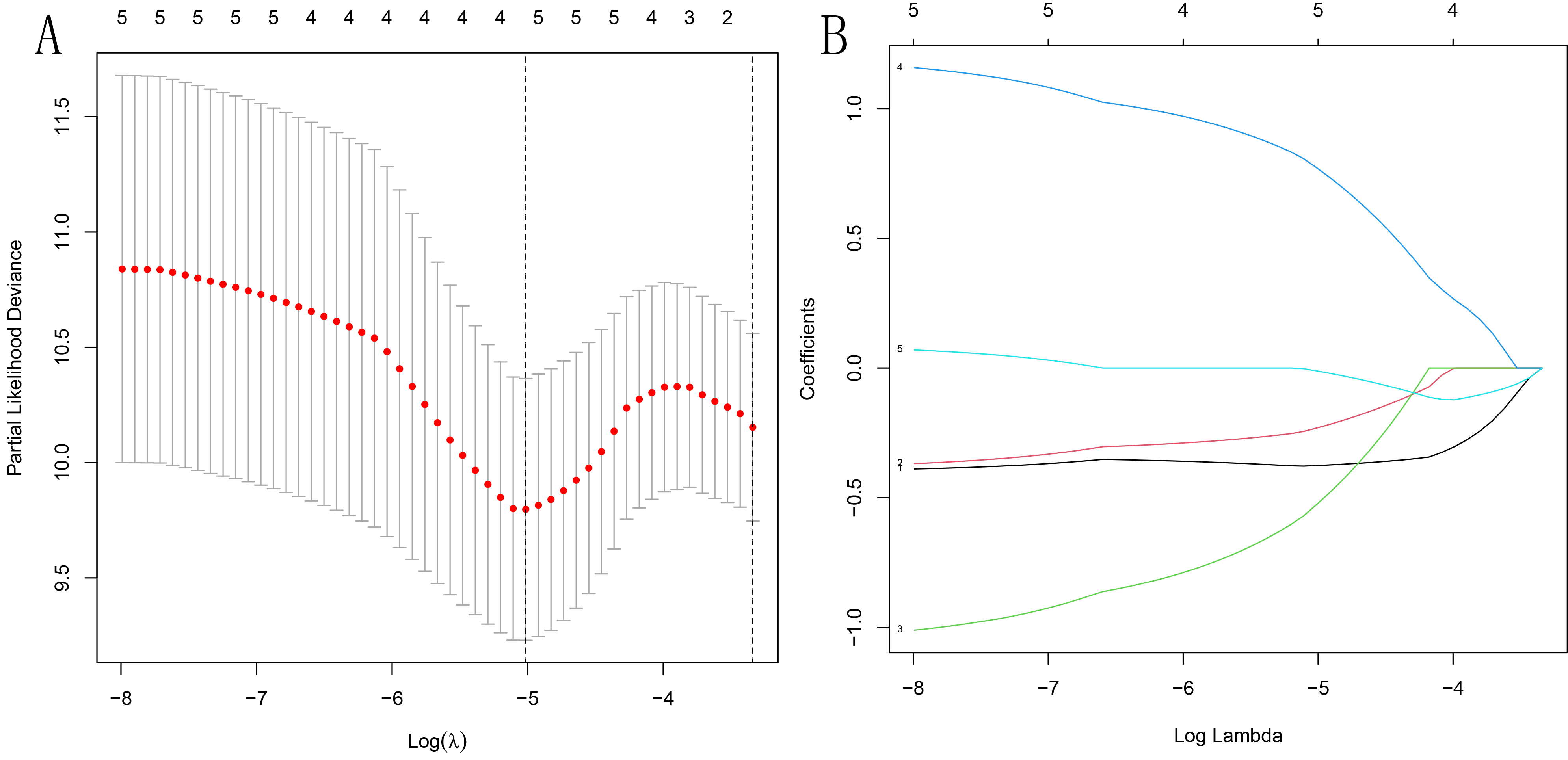

Supplement: Supplementary file 1 [file Image3.JPEG]

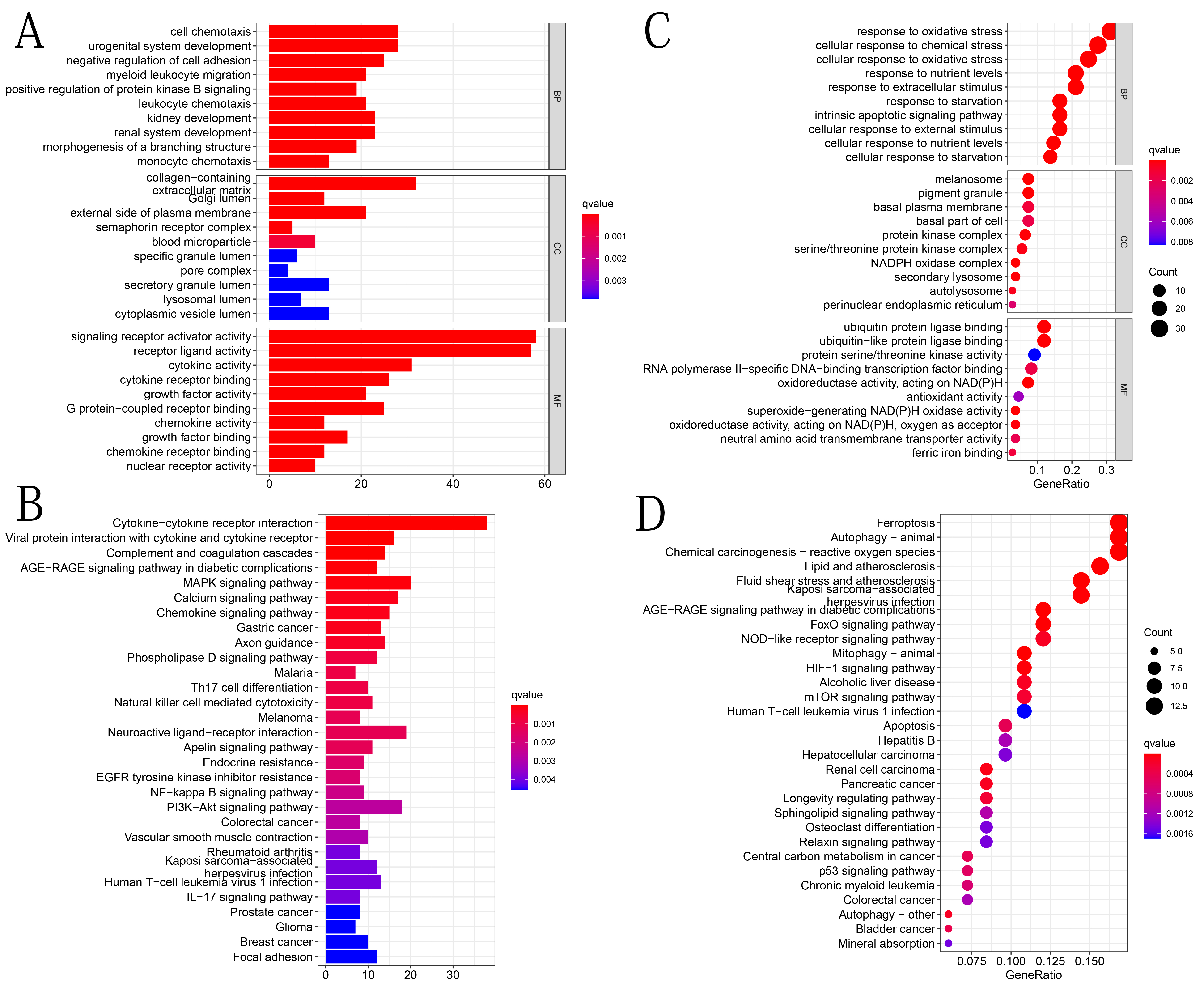

Supplement: Supplementary file 2 [file Image1.JPEG]

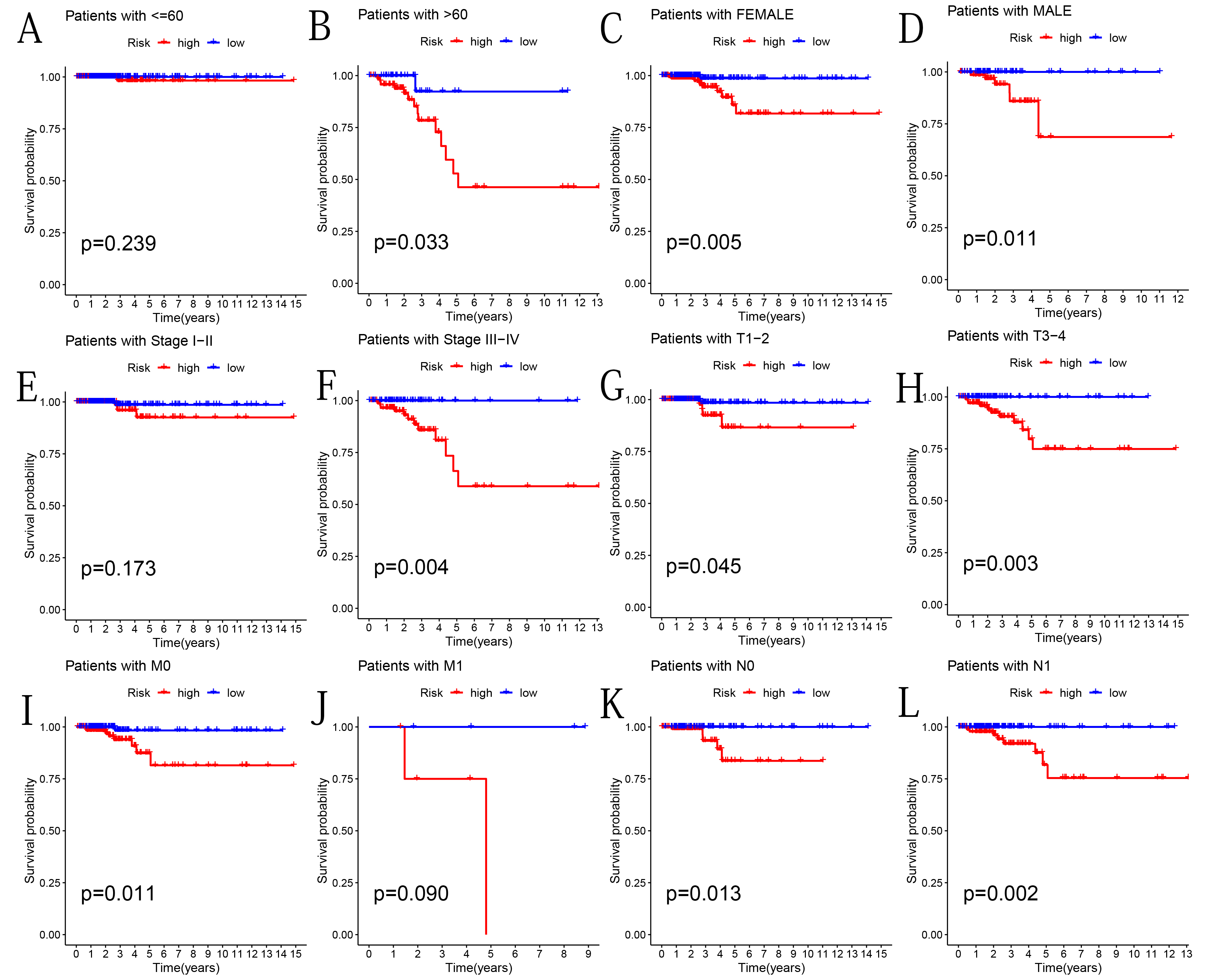

Supplement: Supplementary file 3 [file Image4.JPEG]

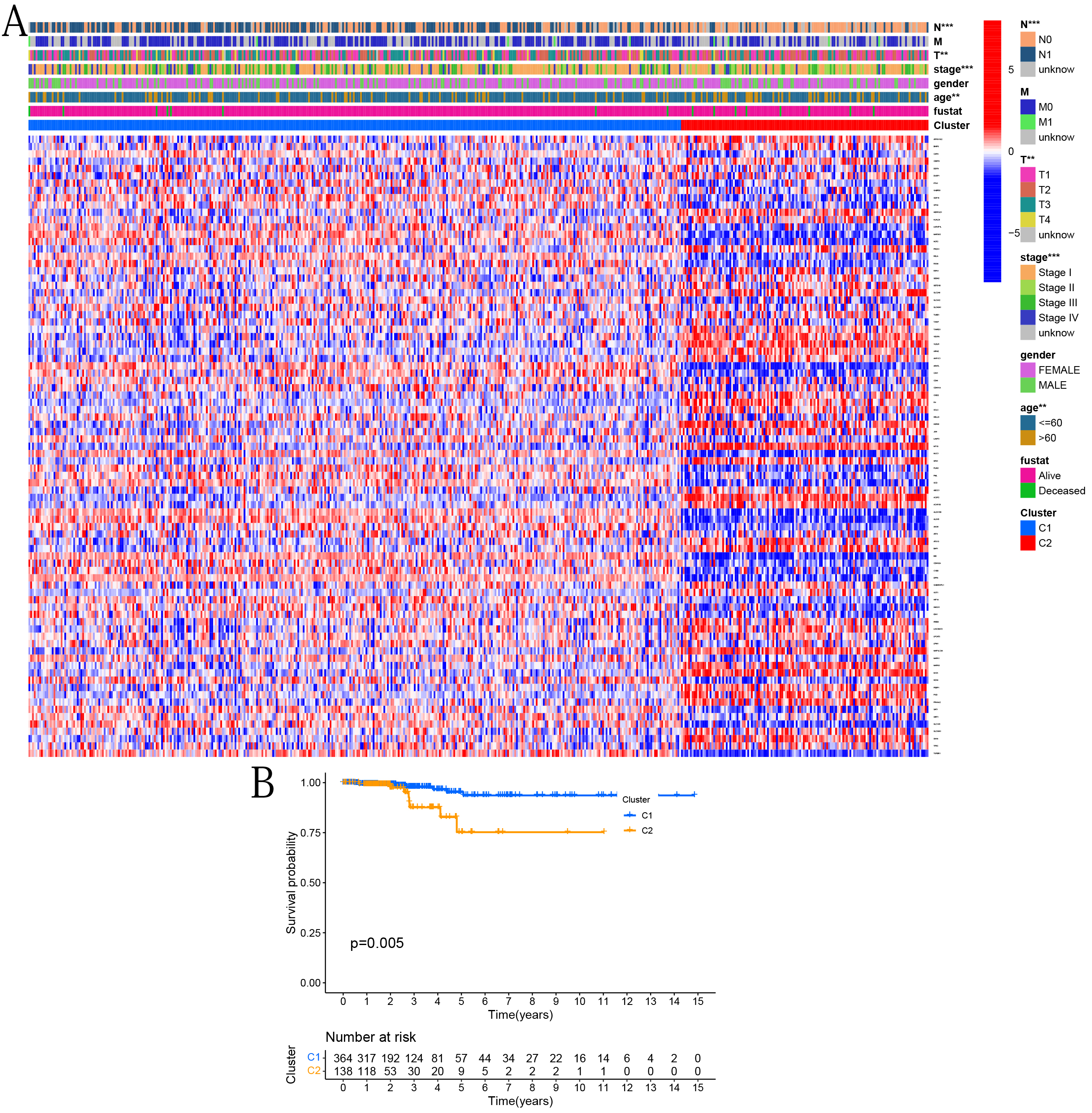

Supplement: Supplementary file 4 [file Image2.JPEG]

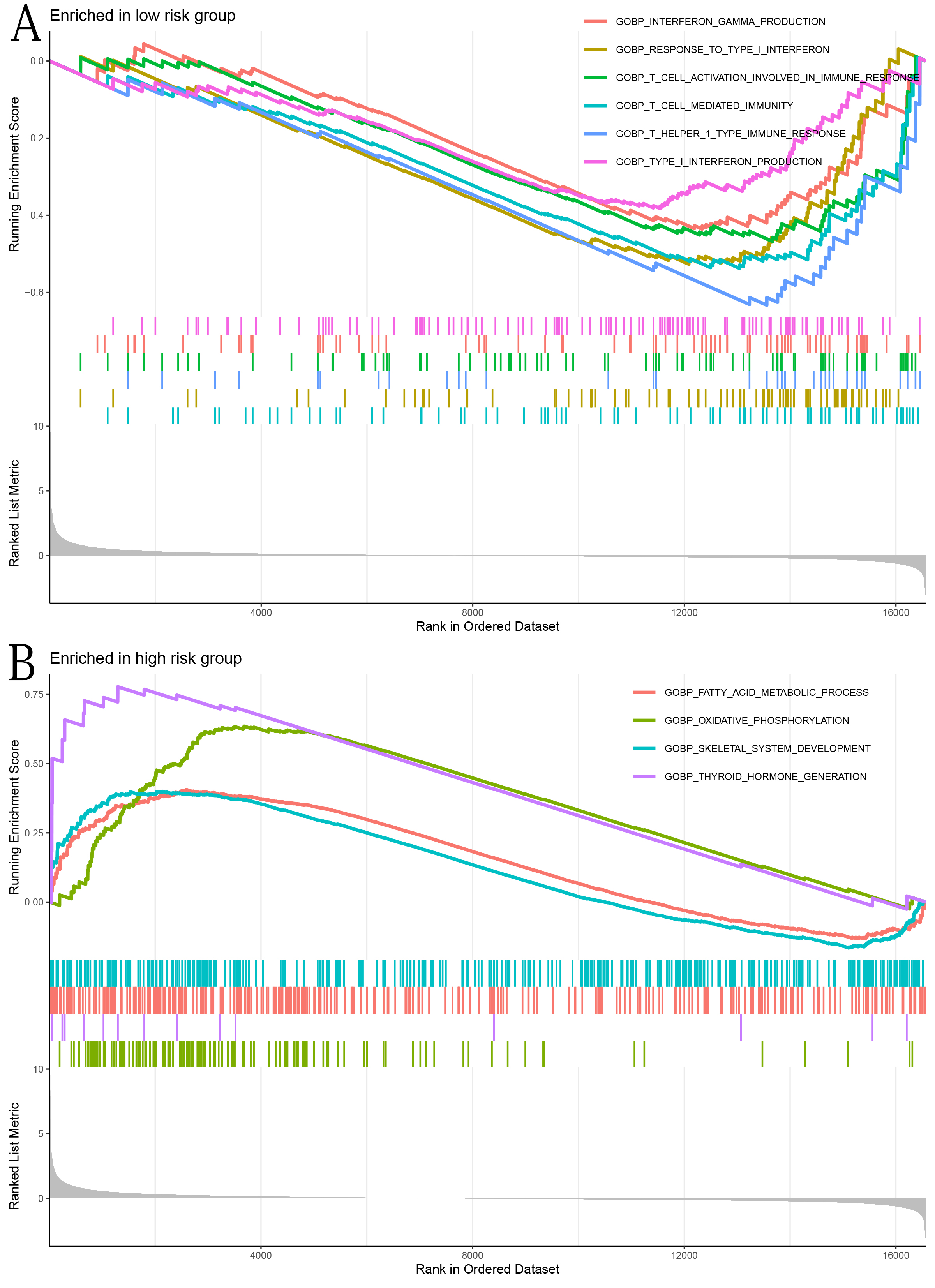

Supplement: Supplementary file 5 [file Image5.JPEG]

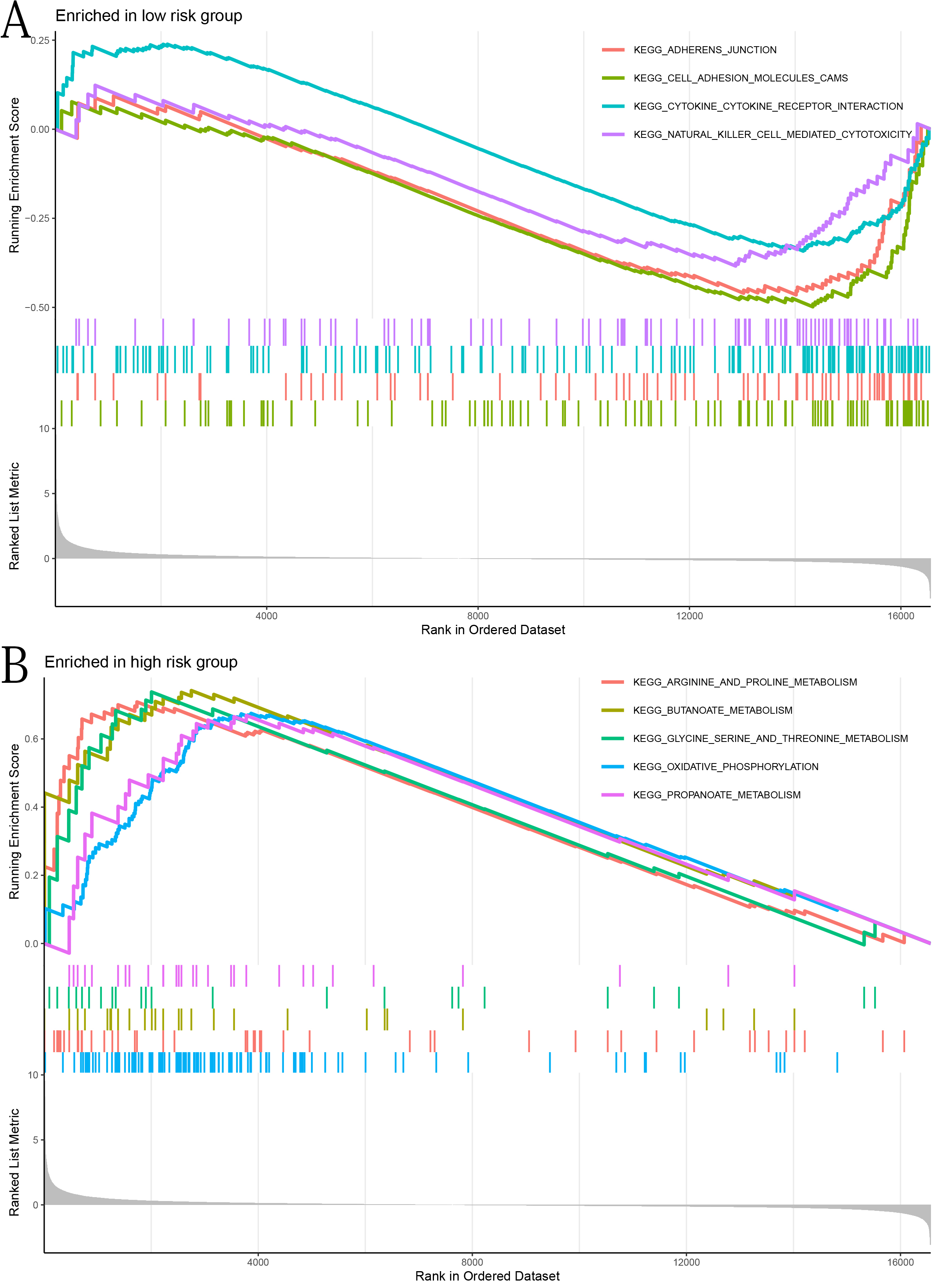

Supplement: Supplementary file 6 [file Image6.JPEG]
